# Genome-wide analysis of the *SWEET* gene family in *Hemerocallis citrina* and functional characterization of HcSWEET4a in response to salt stress

**DOI:** 10.1186/s12870-024-05376-y

**Published:** 2024-07-11

**Authors:** Lihong Cao, Jinyao Wang, Lixuan Wang, Huili Liu, Wenjing Wu, Feifan Hou, Yuting Liu, Yang Gao, Xiaojing Cheng, Sen Li, Guoming Xing

**Affiliations:** 1 College of Horticulture, Shanxi Agricultural University, Taigu, 030801 Jinzhong China; 2 Datong Daylily Industrial Development Research Institute, Datong, 037000 China

**Keywords:** *Hemerocallis citrina*, *SWEETs*, Genome-wide, Abiotic stress, Functional characterization

## Abstract

Sugars will be eventually effluxed transporters (*SWEETs*) have been confirmed to play diverse physiological roles in plant growth, development and stress response. However, the characteristics and functions of the *SWEET* genes in *Hemerocallis citrina* remain unclear and poorly elucidated. In this study, the whole genome of *Hemerocallis citrina* was utilized to conduct bioinformatics analysis and a total of 19 *HcSWEET* genes were successfully identified. Analysis of the physicochemical properties indicated dominant differences among these *HcSWEETs*. A phylogenetic analysis revealed that HcSWEET proteins can be divided into 4 clades ranging from Clade I to IV, where proteins within the same clade exhibited shared conserved motifs and gene structures. Five to six exons were contained in the majority of *HcSWEET* genes, which were unevenly distributed across 11 chromosomes. The gene duplication analysis showed the presence of 4 gene pairs. Comparative syntenic maps revealed that the *HcSWEET* gene family might present more closed homology in monocotyledons than dicotyledons. Cis-acting element analysis of *HcSWEET* genes indicated key responsiveness to various hormones, light, and stresses. Additionally, transcriptome sequencing analysis suggested that most *HcSWEET* genes had a relatively higher expression in roots, and *HcSWEET4a* was significantly up-regulated under salt stress. Overexpression further verified the possibility that HcSWEET4a was involved in response to salt stress, which provides novel insights and facilitates in-depth studies of the functional analysis of HcSWEETs in resistance to abiotic stress.

**Supplementary Information:**

The online version contains supplementary material available at 10.1186/s12870-024-05376-y.

## Background

Growth and development processes are often severely affected when plants are exposed to various abiotic stresses such as drought and salinity due to their sessile characteristics [1, 2]. Sugars, as major sources of carbon skeletons for cell wall and energy metabolism, are crucial signals that regulate the expression of related genes under abiotic stress [3, 4]. Cell-to-cell transport of sugars is mediated by transport proteins. Thus far, three transporters have been identified, including MSTs (monosaccharide transporters), SUTs (sucrose transporters), and SWEETs (sugars will eventually be exported transporters) [5, 6]. Unlike two other transporters, the SWEETs can typically act as bidirectional transmembrane transporter for sugars to cross the biomembrane system along a pH-independent concentration gradient via MtN3/saliva structural domains [7]. Members of the *SWEET* gene family encode membrane proteins consisting of conserved transmembrane (TM) domains linked by a PQ-loop-repeat, which are conserved across eukaryotes and prokaryotes [8, 9]. However, Eukaryotic SWEET proteins generally contain seven transmembrane helices (TMHs), obviously distinguishable from semiSWEETs consisting of 3 TMHs in prokaryotes, which may be correlated to the evolution process of plants [10, 11].

As a transporter identified about sugars recently, the SWEETs are found to be present in all kingdoms of life [5, 9]. SWEET proteins play central roles in plant growth and development such as plant defense, long-distance transport of sucrose, vegetative and reproduction growth, senescence, and stress responses [12–14]. Currently, SWEET proteins have been reported to form four clades (Clade I, II, III and IV) in several plants, furthermore genes belonging to the same clade hold similar structures and functions [8, 15]. Considering the physiological relevance of the fertility of sucrose, the AtSWEET13 sucrose transporter contributes to male fertility restoration [16]. Vacuolar Sugar Carrier *AtSWEET16* overexpression lines promote stress tolerance under non-favorable conditions [17]. OsSWEET11 and OsSWEET15 participate in seed development in rice [18]. Clade III OsSWEETs, which are taken as interaction factors, mediate bacterial blight susceptibility by interacting with OsHMGB1 and OsHsp20L to breed broad-spectrum disease-resistant rice [19]. SlSWEET7a and SlSWEET14 negatively regulate the sugar transport and storage of fruits in tomatoes [20]. MdSWEET9b positively regulates sugar accumulation in apples, providing the foundation for exploring the metabolism between hormone and sugar pathways [21]. GmSWEET15 in soybean mediates sucrose export from the endosperm to the early embryo seed development [22]. CsSWEET17 is activated to positively regulate cold tolerance by interacting with CsLHY in tea plants [23]. Pineapple SWEET10 functions as a potential glucose transporter, and fruit crop yield and quality may be improved by manipulating the glucose transport activity of AcSWEET10 [24]. SWEET11b transports both sugar and cytokinin to maintain normal development in barley grains [25].

Cell membrane transfers chemical signals to the cytoplasm when plants receive stress signals under harsh environmental conditions. Interestingly, for most stresses, the prior exposure can strengthen plants tolerance during subsequent exposures [26]. In the long term, environmental stresses like drought and salinity can activate stress-related response genes of plants to adjust to osmotic pressure [27–30]. Therefore, innovative solutions are inevitable to develop to mitigate soil salinity and drought stress. Sucrose transport, a key physiological process, is impacted by abiotic stresses [31]. Overexpression of *OsMST6* confers improved resilience to drought and salt stress in *Arabidopsis thaliana* [32]. Up-regulation of SUTs may be necessary to maintain phloem transport despite decreased sucrose available under salt and drought stresses in plants [33]. OsSUTs show significant importance in coping with drought and salt stress for rice plants [34]. The increase of soluble sugar contents and tolerances to both drought and salt stress are enhanced by elevating the production of trehalose in *OsTPSP* overexpression lines [35]. OsSWEET13 and OsSWEET15 transporters of Clade III pose critical importance of sucrose translocation and distribution in adaptive responses of rice plants to abiotic stresses [31].

Night lily (*Hemerocallis citrina* Borani) is one of the nutritionally, medicinally, and ornamentally important horticultural perennial crops cultivated worldwide. However, the lack of the night lily genome restrains the progression that molecular mechanisms and gene function analysis are explored. Therefore, as genomic information was released in 2021, new insights will be provided for a deeper understanding of the evolution, development, and biological functions of night lily [36]. As previous results reported, the *SWEET* gene family has become the research focus on responsiveness to abiotic stress. To investigate how night lily responds to drought and salt stress, we performed genome-wide identification of the *SWEET* members. In this study, members of the *HcSWEET* gene family were identified at the genomic level, followed by physicochemical properties, phylogenetic trees, conserved motifs, gene structures, chromosomal positions, cis-acting elements, and collinearity relationships were comprehensively analyzed using bioinformatics methods. Additionally, tissue-specific expression patterns of *HcSWEET* genes, as well as relative expression levels of genes under drought and salt treatments, were analyzed by transcriptome sequencing (RNA-seq). And that RNA-seq data from stress treatments were validated by RT-qPCR. The function of *HcSWEET4a* in stress response was further investigated by the *Agrobacterium*-mediated watermelon transformation method. The research can serve as a promising reference for offering strategies for improving tolerance to abiotic stress in night lily.

## Results

### Identification and characterization of the *SWEET* gene family in night lily

A total of 19 genes that encoded HcSWEET proteins were identified after homology alignment and conservative domain verification. These genes were named *HcSWEET1* to *HcSWEET16* according to their homology with dicotyledons such as *Arabidopsis* AtSWEETs and monocotyledons such as rice OsSWEETs. *HcSWEET* gene characteristics of open reading frame (ORF) size, number of amino acid (AA/aa), protein molecular weight (MW), theoretical isoelectric point (pI), instability index (II), aliphatic index (AI), grand average of hydropathicity (GRAVY), number of predicted TMHs and conserved domain position were analyzed in Table 1. As a consequence, the deduced number of amino acids in the night lily SWEET proteins was from 229 to 299, with the MW varying widely from 25.617 kDa to 32.938 kDa, and the PI index was between 5.49 and 9.66. All proteins ranged from 27.08 to 42.19 on the instability index, from 105.08 to 133.65 on the aliphatic index, and from 0.419 to 0.934 on GRAVY. The majority of predicted TMHs number was seven other than 6 TMHs of *HcSWEET5* and *HcSWEET13a/d*. Coupled with subcellular localization, the secondary structure including α-helix, extended strand, β-turn and random coil were summarized in Supplementary Table S1. Subcellular localization predictions suggested that the majority of *HcSWEET* genes located in the plasma membrane, were also found in the chloroplast, vacuole or cytoplasm (Supplementary Table S1). Considering the transmembrane characteristic, we predicted 3D structural models of 19 HcSWEET proteins using a homology modeling approach, shown in Supplementary Figure S1. The results demonstrated that various properties existed in different *HcSWEET* members.

**Table 1:** Characterization of the *SWEET* gene family in night lily

Gene name	ORF length (bp)	AA(aa)	MW(kDa)	pI	II	AI	GRAVY	THM	MtN3/saliva(PQ -loop repeat) domain position
*HcSWEET1a*	735	244	27190.28	9.26	40.09	112.58	0.666	7	7–95,132–214
*HcSWEET1b*	765	254	28508.65	9.08	27.08	105.08	0.504	7	7–95,132–214
*HcSWEET2*	690	229	25617.54	9.28	35.03	121.16	0.721	7	15–97,134–218
*HcSWEET3*	717	238	26521.72	9.60	42.19	117.52	0.684	7	7–98,132–217
*HcSWEET4a*	774	257	28446.10	9.13	30.78	125.1	0.846	7	11–94,134–218
*HcSWEET4b*	735	244	26987.61	8.95	34.37	133.65	0.95	7	10–98,133–217
*HcSWEET4c*	735	244	26992.56	8.89	37.04	130.82	0.934	7	11–98,133–217
*HcSWEET5*	765	254	28089.69	9.30	30.26	129.61	0.84	6	11–94,134–218
*HcSWEET6*	753	250	27597.16	9.39	35.56	134.04	0.903	7	10–95,134–218
*HcSWEET11*	798	265	29691.26	8.99	36.51	111.09	0.583	7	14–99,133–219
*HcSWEET13a*	747	248	27668.36	8.95	27.33	123.79	0.751	6	12–73,107–188
*HcSWEET13b*	867	288	32084.82	5.51	36.81	117.4	0.554	7	12–82,134–215
*HcSWEET13c*	846	281	31554.43	8.86	34.76	118.9	0.52	7	12–98,134–215
*HcSWEET13d*	747	248	28178.51	8.63	30.77	118.23	0.447	6	1–75,110–196
*HcSWEET14a*	837	278	31378.75	9.23	33.44	124.5	0.626	7	12–99,133–215
*HcSWEET14b*	858	285	32145.67	8.96	29.52	123.12	0.629	7	12–99,133–215
*HcSWEET14c*	858	285	32321.79	8.49	28.01	115.58	0.524	7	12–99,133–215
*HcSWEET15*	870	289	32114.98	5.49	40.11	124.15	0.754	7	12–98,134–215
*HcSWEET16*	900	299	32938.05	9.66	32.26	111.07	0.419	7	8–90,128–211

### Phylogenetic analysis of HcSWEET proteins

**Fig. 1:**
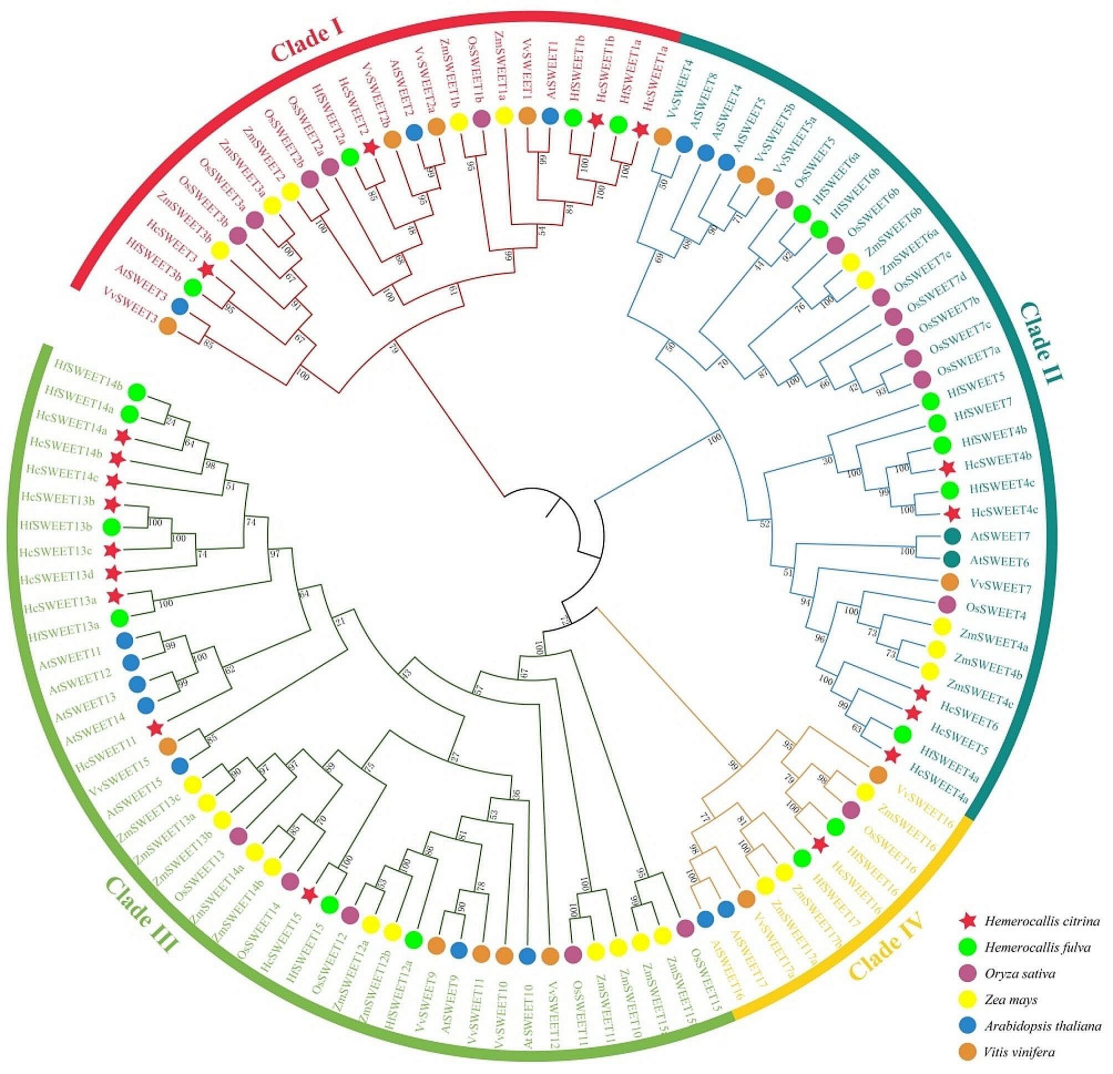
Phylogenetic tree analysis and classification of the SWEETs in night lily and some other plants. Gene members of *Hemerocallis citrina* (Hc), *Hemerocallis fulva* (Hf), *Oryza sativa* (Os), *Zea mays* (Zm), *Arabidopsis thaliana* (At), *Vitis vinifera* (Vv) were classified into 4 clades. A phylogenetic tree was constructed via the TBtools software with 5000 bootstrap replicates

To better understand the evolutionary interrelatedness among these HcSWEET members, we constructed a phylogenetic tree containing SWEETs from six species (*Hemerocallis citrina*, *Hemerocallis fulva*, *Oryza sativa*, *Zea mays*, *Arabidopsis thaliana*, *Vitis vinifera*) based on amino acid sequences using the TBtools software (Fig. 1). SWEET protein sequences of six species were listed in Supplementary Information 1. The results revealed that 19 HcSWEET members were classified into four major clades, namely Clade I, II, III, and IV respectively. Multiple HcSWEETs were tightly clustered with other SWEETs. Among these clades, the member number varied significantly. Clade III contained the most HcSWEET members (9), followed by Clade II (5), I (4), and IV (1), indicating a similar distribution pattern and gene function to these of other species.

### Conserved motif composition, domain, and gene structure analysis of *HcSWEET* genes

**Fig. 2:**
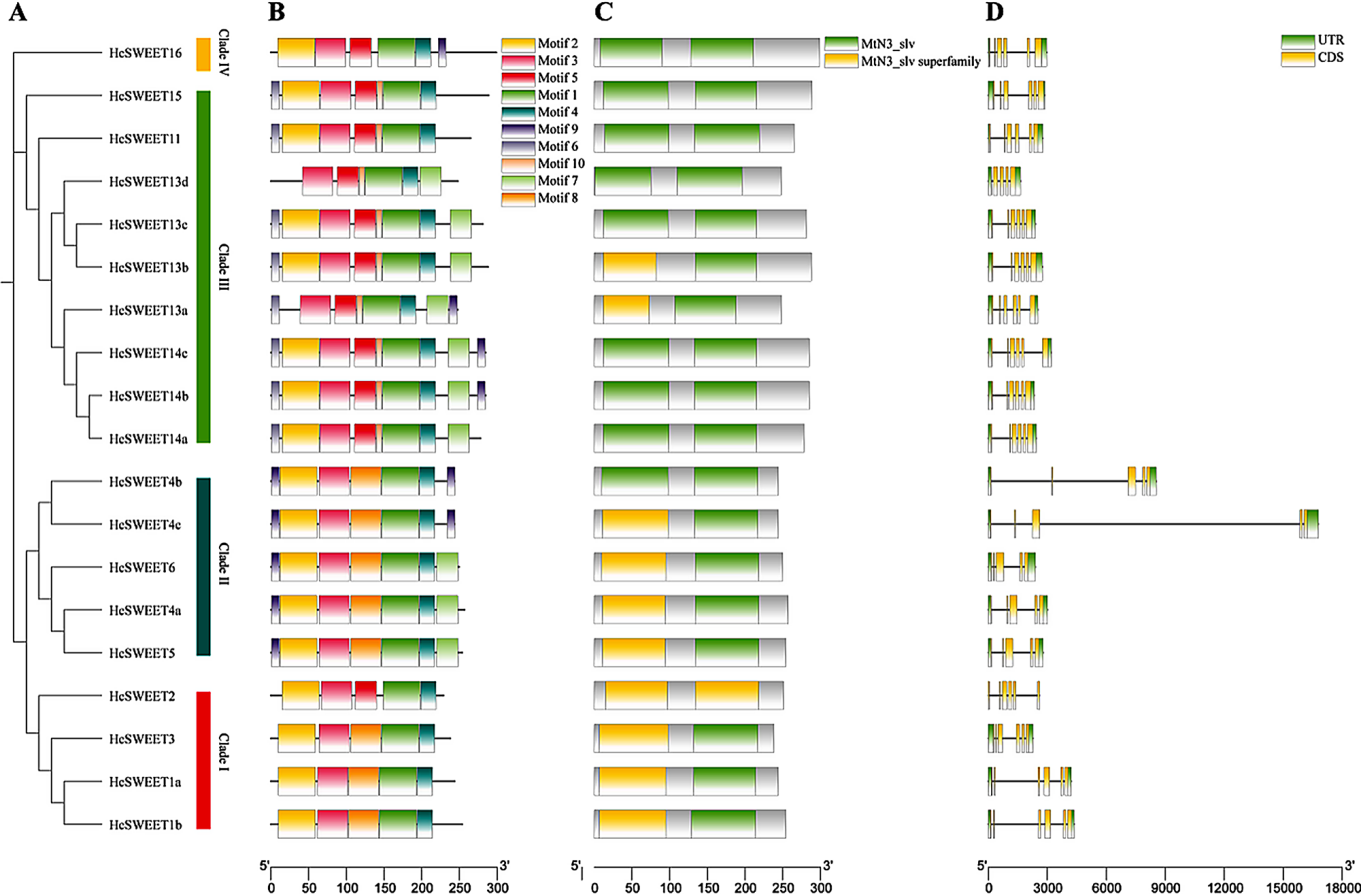
Constructed phylogenetic tree, conserved motif, conserved domain, and gene structure analysis of SWEETs in *Hemerocallis citrina*. (**A**) Intraspecific evolutionary tree of 19 HcSWEET members. (**B**) The compositions and distribution of *HcSWEETs* conserved motifs. (**C**) The conserved functional domains of HcSWEETs. (**D**) Gene structures of *HcSWEET* genes

According to the results from phylogenetic analysis, we divided the identified 19 HcSWEET proteins into 4 clades (Fig. 2A). Ten different conserved motifs were identified by analyzing amino acid sequences of HcSWEET proteins using the MEME online tool (Fig. 2B). The number and distribution of these motifs showed variations in different proteins. Each protein had 5–7 motifs, in which Motif 1, 3, and 4 were found in all proteins, moreover, different clades had distinct motifs. Motif analysis suggested that the *HcSWEET* gene family showed both conservation and differences, which might result from the diversity of protein functions within clades. Conserved domain analysis revealed that HcSWEET proteins possessed domains consisting of MtN3_slv and MtN3_slv superfamily (Fig. 2C), implying high conservation during evolution. In addition, to understand the structural compositions of *HcSWEET* genes, a structural map including the untranslated region (UTR) and coding sequence (CDS) was constructed based on the genome sequence (Fig. 2D). The number of CDSs ranged from 5 to 6. Coding sequences of 19 *HcSWEETs* were displayed in Supplementary Information 2. Gene structure analysis suggested that members within the same evolutionary clade exhibited similar gene structures.

### Chromosomal localization, gene duplication, and collinearity analysis of *HcSWEETs* in *Hemerocallis citrina*, *Arabidopsis thaliana* and *Oryza sativa*

**Fig. 3:**
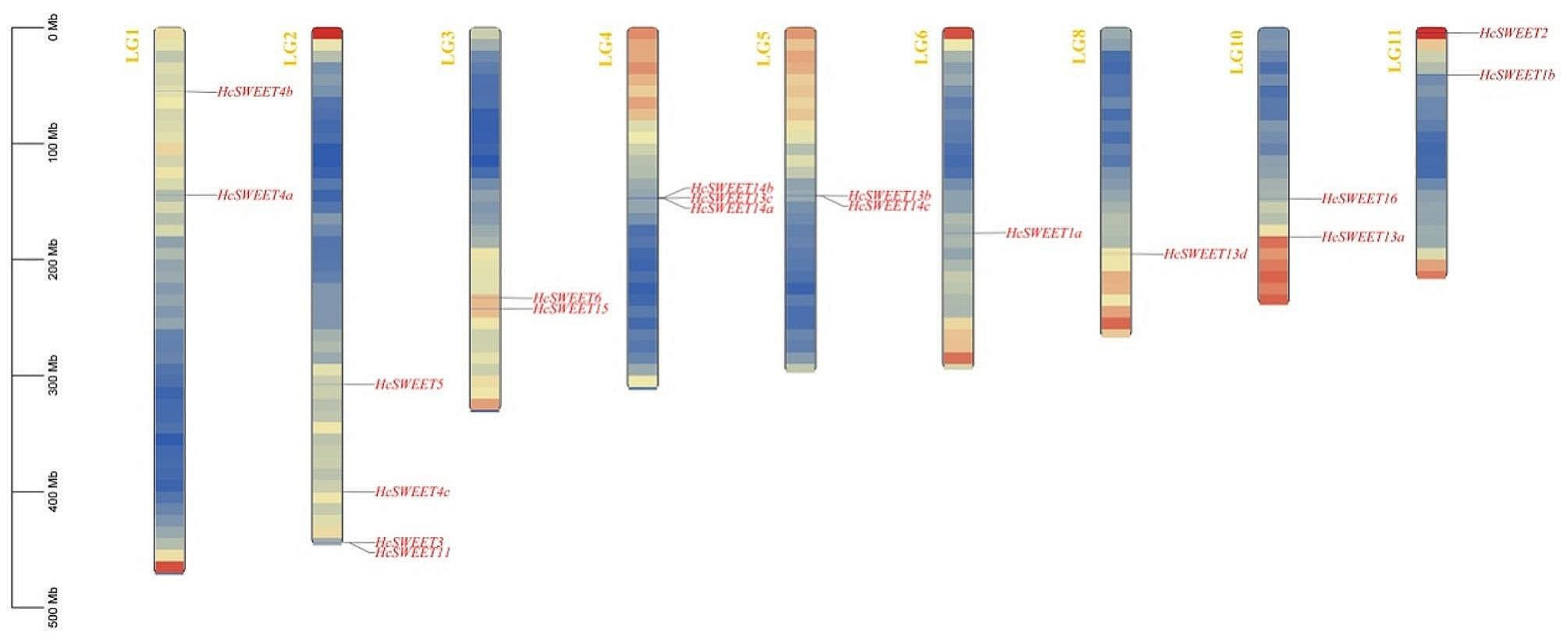
Chromosome distributions of *SWEET* genes on night lily (LG1-11). All *HcSWEET* genes named are displayed on night lily LGs, with the LG number marked at the edge of each strip. The 0-500 Mb scale represents chromosome length. The lines inside the LGs represent gene density

To investigate the chromosomal localization of *HcSWEET* genes, we analyzed the distribution of identified 19 genes on night lily chromosomes (Fig. 3). The distributions of these genes were determined via the night lily genome reported in 2021 [36]. The results showed that 19 genes were evenly dispersed across 9 of 11 chromosomes (LGs) with gene counts ranging from 1 to 4 in total. Notably, the largest number of genes were located in LG2, in contrast, LG6 and LG8 hosted only one gene and no gene was located in LG7 and LG9.

**Fig. 4:**
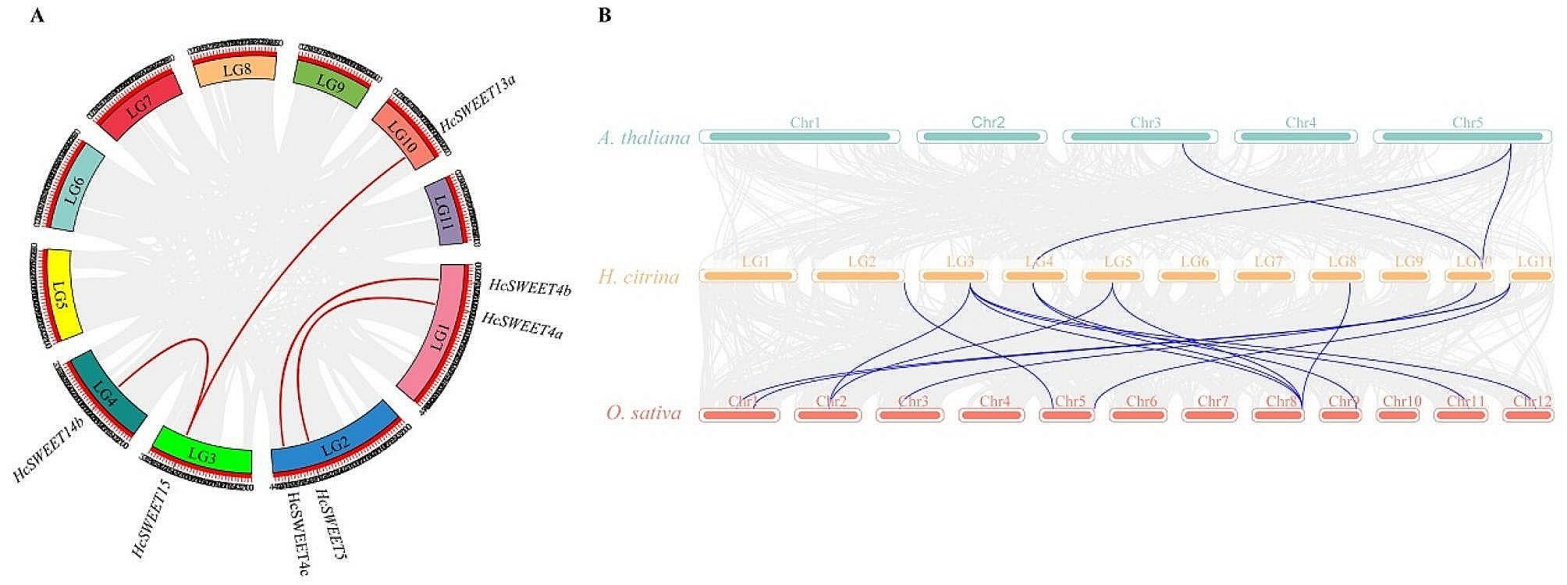
Collinearity of *HcSWEET* gene pairs. (**A**) Chromosome location and inter-chromosomal relationships of *SWEETs* in *Hemerocallis citrina*. The identified duplication events are marked by red lines. The LG number is labeled within different colored rectangles. (**B**) Collinearity analysis between the *SWEETs* of *Hemerocallis citrina* (*H. citrina*), *Arabidopsis thaliana* (*A. thaliana*), and *Oryza sativa* (*O. sativa*)

To set forth whether gene duplication events forced the evolution of the *HcSWEET* gene family, an intra-species collinear analysis was performed (Fig. 4A). Among all gene pairs of the night lily genome, four pairs of duplicated segments were identified in the *HcSWEETs*, implying that segmental duplication events were likely to contribute to genetic diversity. Subsequently, we constructed inter-species syntenic maps of *Hemerocallis citrina* with other two representative species including monocotyledon *Oryza sativa* and dicotyledon *Arabidopsis thaliana* to identify orthologous genes through utilizing TBtools (Fig. 4B). It was observed that seven genes exhibited collinearity relationships with two genes in *A. thaliana* and ten genes in *O. sativa*. These results suggest that *HcSWEET* genes likely share more similarities in function with *OsSWEET* genes in *O. sativa*.

### Identification and distribution of cis‑regulatory elements in *HcSWEET* promoter

**Fig. 5:**
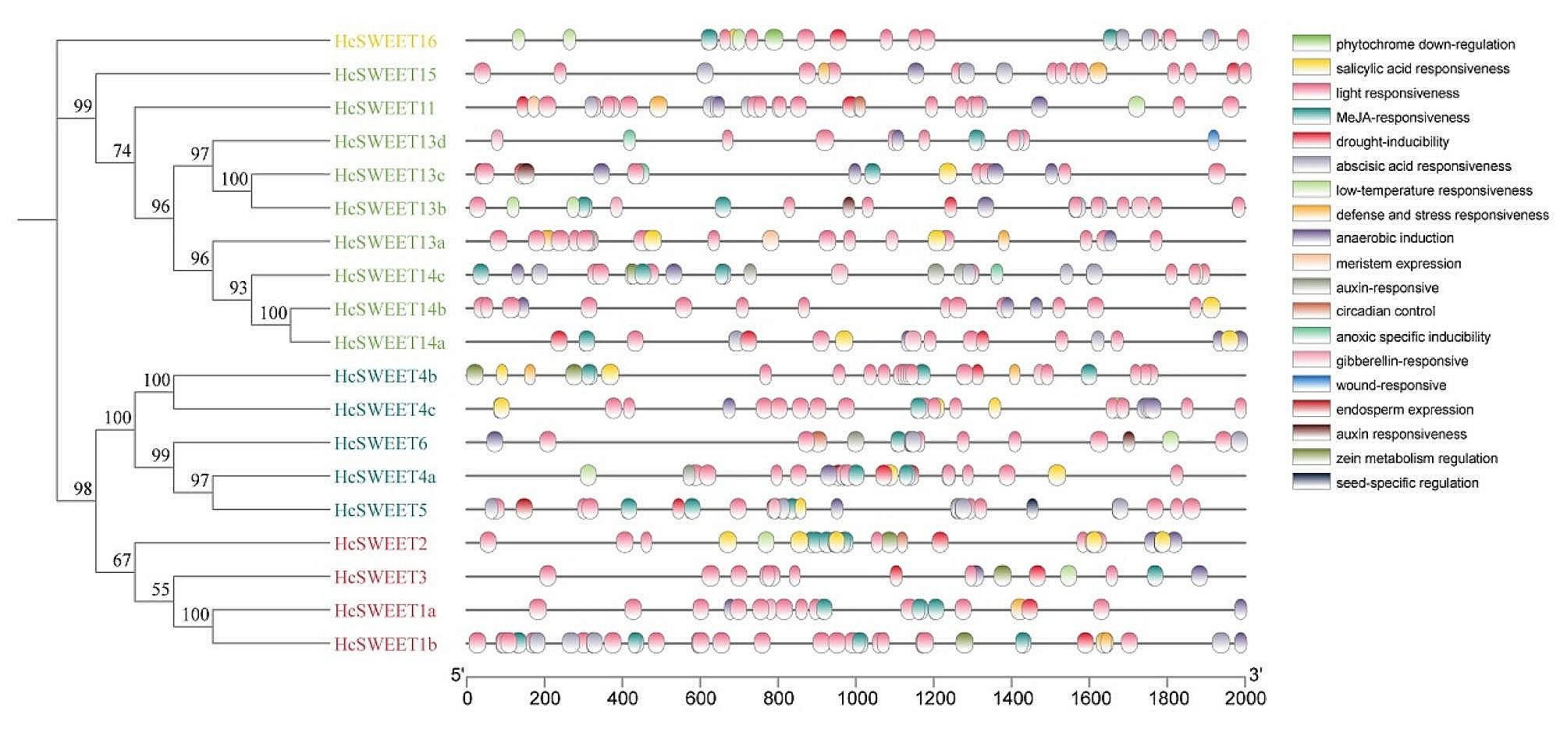
Distributions of cis-acting elements of the *HcSWEET* promoter

We specifically extracted 2000 base pairs upstream of the *HcSWEET* genes transcription start codon to explore their potential biological functions through the database named PlantCARE online. As shown in Fig. 5, a total of three types of main responsive elements were identified, hormone responses, plant growth and development, and responses to abiotic stress respectively. Among them, 6 to 26 light-responsive elements were found in 19 genes, indicating that the majority of *HcSWEET* genes may be regulated by light signaling. Moreover, all 19 genes contained hormone-related and development-related cis-acting elements. Hormone-related elements were referred to as MeJA, salicylic acid, abscisic acid, auxin, and gibberellin response elements. In contrast, development-related elements contained zein metabolism regulation, anaerobic induction, cell cycle regulation, seed-specific regulation, circadian control, flavonoid biosynthesis, endosperm, and meristem expression, anoxic specific inducibility and phytochrome down-regulation response elements. Additionally, *HcSWEET* genes other than *HcSWEET4c*, *HcSWEET13d*, and *HcSWEET14b* had cis-acting elements linked to abiotic stress, involving defense and stress, drought, and low-temperature responsive elements. *HcSWEET13d* also contained the wound-responsive element. Overall, various types of cis-elements were contained in the promoter regions of 19 *HcSWEETs*, indicating their possible involvement in diverse biological processes, environmental stresses, and regulatory pathways.

### Expression patterns of *HcWEET* genes across different tissues and phenotype observations under drought and salt stress

**Fig. 6:**
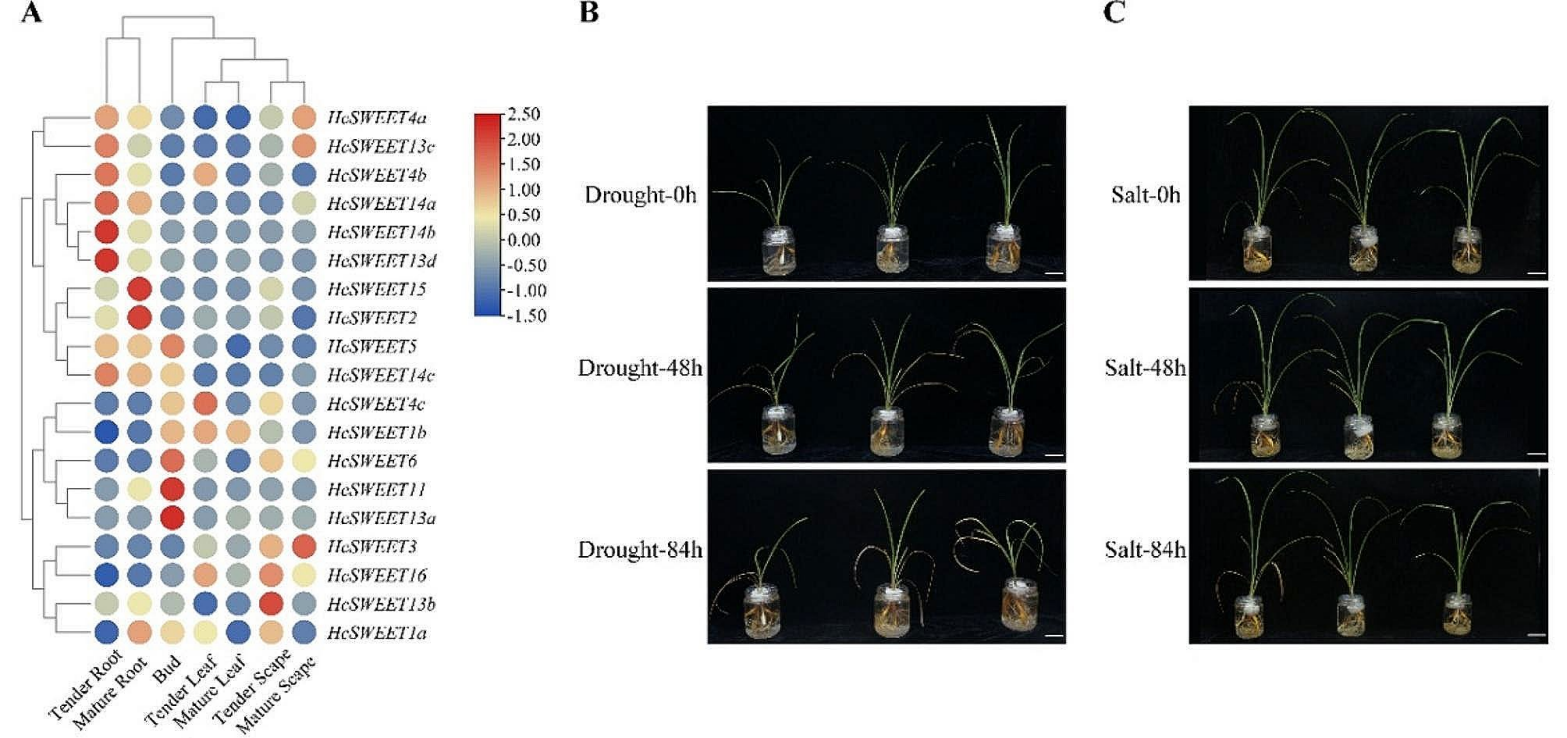
Expression dynamics of the *HcSWEET* genes in different tissues of night lily and phenotypes under different abiotic stresses. (**A**) Expression heatmap of *HcSWEETs* in the tender root, mature root, bud, tender leaf, mature leaf, tender scope, and mature scope. (B, C) The phenotypic observations of night lily under drought stress (**B**) and salt stress (**C**)

To further analyze the possible roles of *HcSWEET* genes in abiotic stress, 19 genes were analyzed for relative expression profiles in different tissues and developmental stages (tender root, mature root, bud, tender leaf, mature leaf, tender scape, and mature scape) (Fig. 6A). As listed in Supplementary Table S3, RNA-seq results of tissue-specific expression revealed that most *HcSWEETs* in Clade II (*HcSWEET4a*, *HcSWEET4b*, *HcSWEET5*) and III (*HcSWEET13c*, *HcSWEET13d*, *HcSWEET14a*, *HcSWEET14b*, *HcSWEET14c*, *HcSWEET15*) exhibited the higher transcript levels in roots than the other organs. Among them, *HcSWEET4a* and *HcSWEET13c* were simultaneously in a related highly expression in the scape. Except these genes, *HcSWEET2* in Clade I was expressed especially and abundantly in roots throughout the developmental process.

The transporters of the *SWEET* gene family in *Arabidopsis thaliana* have been reported to increase root proliferation by enhancing sucrose transport from shoot to root during abiotic stress [37]. Therefore, we carried out stress experiments to determine whether the case was suitable for *HcSWEET* genes in night lily. We treated 30 d seedling roots with drought stress for 0 h, 24 h, 48 h, 72 h, and 108 h, and identically salt stress for 0 h, 24 h, 48 h, 72 h, and 96 h. As was displayed in Fig. 6B and C, night lily plants primarily showed a slight symptom of loss of water when treated for 24 h. After treatment for 48 h, leaves presented severe yellow and rotten roots were generated.

### Expression pattern analysis of *HcSWEETs* under drought and salt stress

**Fig. 7:**
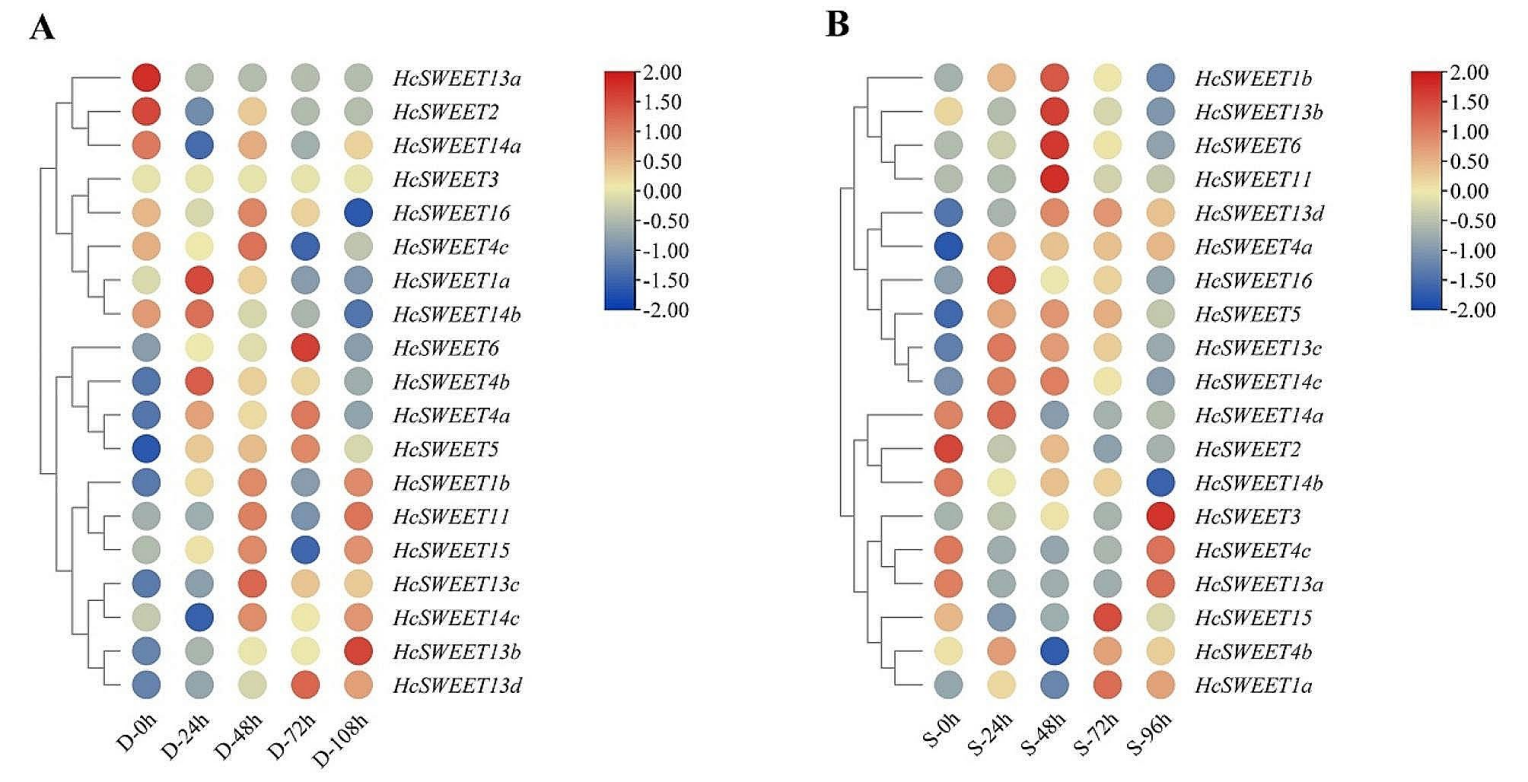
Expression heatmaps of *HcSWEETs* under drought (**A**) and salt (**B**) stress based on RNA-seq data

Based on the phenotypes observed in night lily plants treated by drought and salinity, roots were collected, followed by RNA-Seq analysis (Supplementary Table S4 and S5). Depicted in Fig. 7A, expressions of genes including *HcSWEET1b*, *HcSWEET4a*, *HcSWEET4b*, *HcSWEET5*, *HcSWEET13b*, *HcSWEET13c*, and *HcSWEET13d* were induced compared with the control group (0 h) under drought stress. On the contrary, *HcSWEET2*, *HcSWEET13a* and *HcSWEET14a* expressed in a down-regulation trend. Relative expression levels of two genes (*HcSWEET1a*, *HcSWEET14b*) first increased and then decreased, while *HcSWEET14c* exhibited a contrary expression pattern.

We further investigated expression levels of *HcSWEETs* under salt treatment (Fig. 7B). Five *HcSWEET* genes (*HcSWEET4a*, *HcSWEET5*, *HcSWEET11*, *HcSWEET13c*, *HcSWEET13d*) showed significantly elevated expression levels. By contrast, the expressions of *HcSWEET2*, *HcSWEET6*, *HcSWEET13b*, and *HcSWEET14b* increased during different salt stresses. After salt treatments, there were 4 genes (*HcSWEET1b*, *HcSWEET14a*, *HcSWEET14c*, and *HcSWEET16*) initially up-regulating, subsequently decreasing again in expression levels. *HcSWEET4c*, *HcSWEET13a*, *HcSWEET15* went down and then up. The various expression types showed that functional differences were likely to exist in *HcSWEET* genes in response to abiotic stresses.

**Fig. 8:**
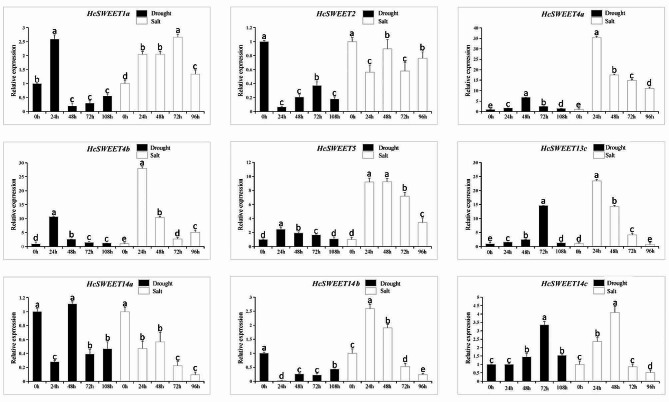
Expression patterns of 19 *HcSWEET* genes after drought and salt treatment. The different lowercase letters indicate significant differences by t-test (*p* < 0.05)

To unveil the potential functions of *HcSWEET* genes in responding to different stresses including drought and salinity, nine *HcSWEET* genes showing significant responses were selected and RNA-Seq results were further confirmed using RT-qPCR experiments (Fig. 8). *HcSWEET4a*, *HcSWEET4b*, and *HcSWEET5* showed an up-regulation in expressions under two stresses, notably, the expressions of *HcSWEET4a* and *HcSWEET5* significant increased after salt stress, suggesting the potential involvement of *HcSWEET4a* and *HcSWEET5* in salt stress response. *HcSWEET2* exhibited a pattern of decreased expression compared with 0 h. Given whole wavelike patterns, the expressions of *HcSWEET1a*, *HcSWEET4a*, *HcSWEET4b*, *HcSWEET5*, and *HcSWEET13c* had a sharp increase at the time point of 24–72 h, but decreased in 96–108 h throughout stresses. Overall, our analysis revealed that expression trends of 19 genes were consistent with those of the RNA-Seq and the majority of *HcSWEETs* presented a dynamic response pattern.

### Subcellular localization of HcSWEET4a and HcSWEET5

**Fig. 9:**
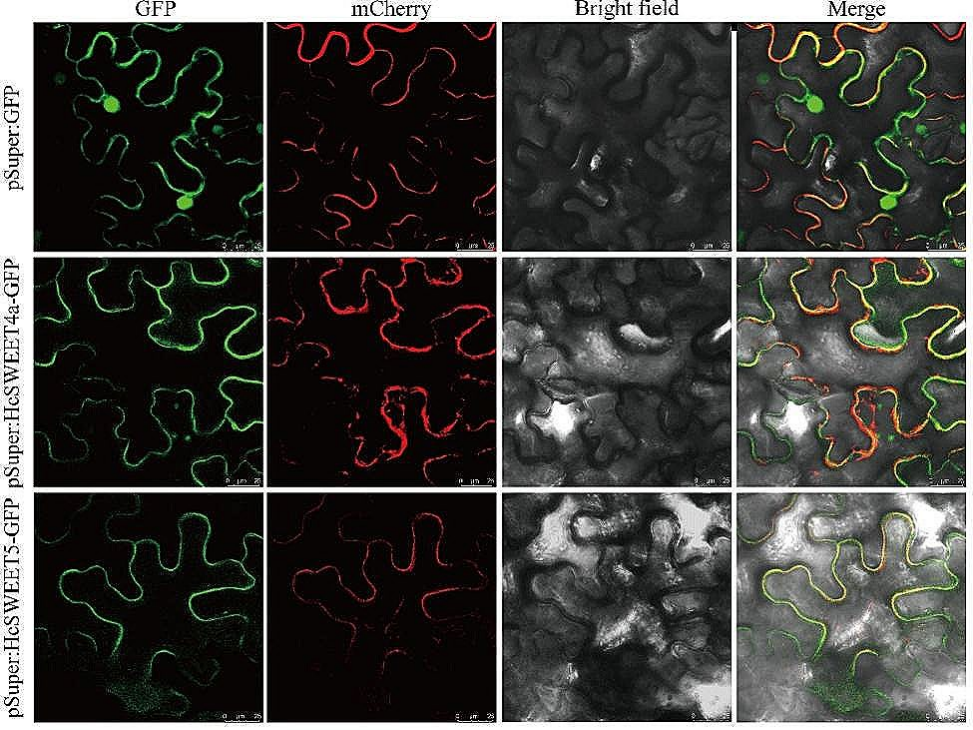
Subcellular localization of HcSWEET4a and HcSWEET5. pSuper: GFP represents the negative control. The second panel represents a positive marker for the plasma membrane. The rightmost panel indicates the fusion of green fluorescence, red fluorescence, and bright field. Bar = 25 μm

HcSWEET4a and HcSWEET5 were predicted to function in the plasma membrane (Supplementary Table S1). To evaluate the validity of results, we performed co-expressed experiments in *N. benthamiana* leaf cells (Fig. 9). The fusion constructs pSuper: HcSWEET4a-GFP and pSuper: HcSWEET5-GFP were co-expressed with the plasma membrane marker (mCherry). Excluding empty vectors pSuper-GFP, GFP fluorescent signals were all exclusively observed in the plasma membrane. Results indicated that HcSWEET4a and HcSWEET5 belonged to plasma membrane-localized proteins.

### Overexpression of *HcSWEET4a* in watermelon and response to salt stress

**Fig. 10:**
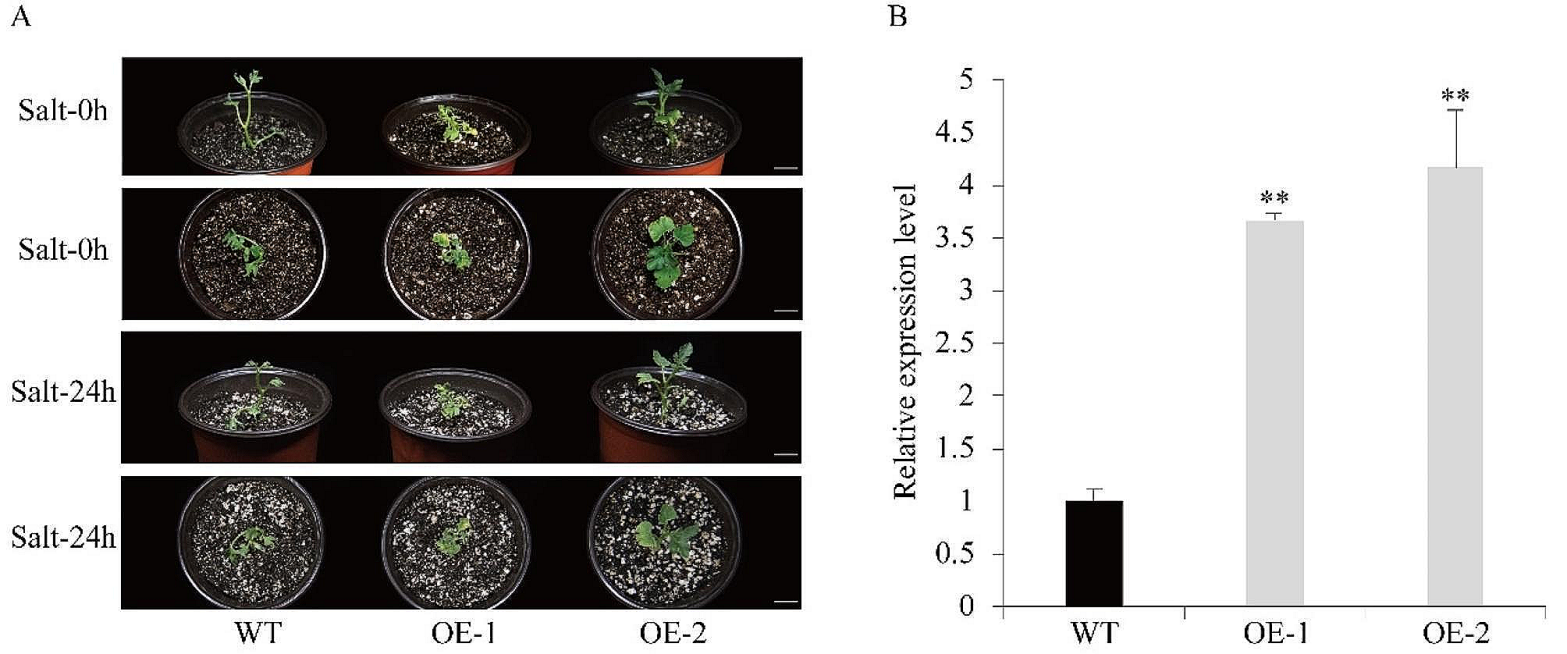
Phenotype observations of *HcSWEET4a* overexpression lines under salt treatment. (**A**) Phenotype changes of transgenic lines (OE-1, OE-2) after treated for 0–24 h with NaCl (150 mM). Bar = 17 mm. (**B**) The relative expression levels of *HcSWEET4a* in OE-1 and OE-2 lines. The significance of difference is marked by two asterisks (*p* < 0.05)

Previous experiments have implied that both *HcSWEET4a* and *HcSWEET5* exhibited significant up-regulation under salt stress. To further interpret the function of HcSWEETs in response to salt stress. We ectopically overexpressed alternative *HcSWEET4a* in watermelon through *Agrobacterium*-mediated transformation. Two transgenic lines (OE-1, OE-2) were obtained and expression levels were 3.67–4.18 times higher than the wild-type (WT) watermelon plant (Fig. 10B). Under NaCl treatment (150 mM) for 24 h, transgenic plants exhibited slightly curled leaf margins, while WT plants have been almost wilted (Fig. 10A). Phenotype observations further showed the up-regulation of *HcSWEET4a* might improve tolerance to salinity of plants.

## Discussion

*Hemerocallis citrina*, belonging to Liliaceae *Hemerocallis*, is an important specialty crop widely cultivated around the world. With continuously expanded consumption patterns, night lily tends to be a model vegetable species for understanding deeply of perennial plant growth and development [38, 39]. Thereby, its tolerance to abiotic stress has become one of most key elements which influence production and quality. *SWEET* genes are plant-specific and taken as an essential class of sugar transporters in multiple plants [14, 40]. No studies were reported on the genome-wide identification of *HcSWEETs* or their function validation in perennial night lily previously. The present study investigated the response patterns of *HcSWEET* genes through genome-wide and expression analysis under drought and salt stress, aiming at promising new insights into enhancing stress resistances of night lily.

The study isolated 19 functional *HcSWEETs* successfully (Table 1), whose characteristics were similar to those reported in other plants especially *HfSWEETs* [41]. Secondary (Supplementary Table S1) and tertiary (Supplementary Figure S1) structure of HcSWEET proteins were predicted, revealing potential functions and interactions between proteins [42]. HcSWEETs fell into four phylogenetic clades by bioinformatics analysis, which were uniform with HfSWEETs, AtSWEETs, VvSWEETs, and LcSWEETs [5, 41, 43, 44]. There are not only similarities but also discrepancies in the members’ number of the same clade, for instance, as was shown in (Fig. 1), nine members were found in Clade III contrast sharply to only one in AtSWEET and OsSWEET [45–48]. Phylogenetic analyses were supported by analyses of conserved motifs and gene structures. Conserved motif analysis indicated that most HcSWEETs harbored seven conserved transmembrane domains (TMDs), while genes within each clade also shared more unique motifs (Fig. 2A-B). Above conclusion is consistent with *Arabidopsis thaliana*, *Solanum lycopersicum*, and *Oryza sativa* [5, 49, 50]. Gene structure model demonstrated that *HcSWEETs* within each clade were similar in an exon-intron organization (Fig. 2D). For instance, each member of Clade I, III (except *HcSWEET13d*), and IV harbors 6 exons, whereas genes in Clade II contain 5 exons. Previous relative report in *Oryza sativa* indicated the loss-of-exon rate is larger than the gain-of-exon rate after undergoing segmental duplication [51]. Therefore it is proposed that all clades except Clades II might contain the original ancestor *HcSWEET* genes, and genes in Clade II might result from gene duplication and divergence.

*HcSWEET* genes were uniformly located in 9 chromosomes (Fig. 3). Intra-species collinear analysis identified four pairs of duplicated segments (Fig. 4A), and inter-species syntenic maps identified seven homologous genes in *Arabidopsis thaliana* but two in *Oryza sativa* (Fig. 4B). Therefore, we speculated that tandem or segmental duplication might result in species evolution by gene family expansion, consistent with other plant species reported [9, 48, 52]. The promoters of *HcSWEETs* mainly contain stress response elements, growth and development-related elements, hormone response elements (Fig. 5), suggesting that *HcSWEET* genes are indispensably in the process of various biological activities and coping with abiotic stresses of night lily. At the same time, some previous reports establish a theoretical foundation for further exploring gene functions of *HcSWEETs* [53–62].

SWEETs in the same or different species may have different cellular localizations [5, 17, 63]. AtSWEET4 in *Arabidopsis* is localized in the plasma membrane, mediating sugar transport to axial sinks as a hexose facilitator [64]. OsSWEET4 and ZmSWEET4c are located in the basal endosperm transfer layer membrane, transferring hexoses across BETL to sustain the development of large endosperm in cereal grains [65]. SWEET4 in grapevine as a glucose transporter, was also identified as a plasma membrane protein [44]. Sugar transporter gene *PbSWEET4* in pear, localizing in the plasma membrane, causes the reduction of sugar content and leads to early senescence of leaves [66]. In our present study, HcSWEET4a and HcSWEET5, falling in Clade II, were localized in the plasma membrane (Fig. 9). AtSWEET11 and close paralog AtSWEET12 localize to the plasma membrane of the phloem, suggesting the same localization also may exist in different clades [67].

At present, the SWEET family of transporters has been characterized as one of the key players in sugar translocation in higher plants [5, 6]. The major types of translocated sugar and their regulation in partition are necessary concerning plant development and stress responses [68, 69]. In divided four clades, genes in Clade I and II prefer hexose, Clade III sucrose, and Clade IV SWEETs likely fructose transporters in *Arabidopsis* [17, 67]. Expression patterns of genes are tightly correlated with functions, thus, tissue-specific analysis can be used to predict biological functions [70]. In this study, genes in the same clade tended to present the same expression pattern (Fig. 6). In Clade II, *HcSWEET4a*, *HcSWEET4b*, and *HcSWEET5*, highly expressing in roots except for *HcSWEET4c* and *HcSWEET6* in buds, might play central roles in mediating stress response as reported in *Arabidopsis* [64]. Our finding indicated that similar biological functions are performed more possibly in the same subfamily.

SWEETs in cabbage and *Medicago truncatula* have been reported in response to chilling stress [59, 71]. MaSWEETs are involved in abiotic/biotic stress responses in bananas [45]. *ZmSWEET* gene in maize responds to abiotic stress [72]. The *SWEET* gene family in wheat response widely to abiotic stress [73]. Our RNA-seq data preliminary showed that expression levels of most *HcSWEETs* first increased after treatments for 24 h compared with 0 h, then changed towards different trends (Fig. 7), RT-qPCR assay further verified the expression levels of some differential expression genes (DEGs) in the *HcSWEET* gene family to ensure the reliability (Fig. 8). These experiments jointly suggested that most HcSWEETs might respond to drought and salt stress in different regulatory pathways with functional redundancy.

The members of the *SWEET* gene family may act as sugar transporters exposed under adverse environmental conditions [74, 75]. To elucidate precise roles of *HcSWEET4a* in night lily, ectopic expression of *HcSWEET4a* was conducted in watermelon. The results showed that up-regulated expression of *HcSWEET4a* in transgenic lines was distinct compared with WT plants (Fig. 10A). Phenotype observations indicated transgenic lines have greater resistance against salinity than WT plants. All data integrated in our present study, It is concluded that HcSWEET4a enhances salt tolerance by positively regulating sugar transport. However, the internal regulatory network remains poorly elaborated.

## Conclusions

In summary, our present study first analyzed all extracted SWEET sequences in *Hemerocallis citrina*. Nineteen *HcSWEET* genes were selected as members comprehensively characterized, including basic properties, evolutionary relationships, conserved motifs, protein domains, gene structure analysis, chromosomal localization, gene duplication, and collinearity analysis. The phylogenetic tree classified the *HcSWEET* gene into four clades. Each clade had similar motif compositions and gene structures. Tissue-specific expression patterns of *HcSWEETs* and DEGs under drought and salt treatments suggested the function diversity and potential response mechanism to environments. Ectopic expression in watermelon further assured the response of HcSWEET4a to salt stress. Our research lays a foundation for casting light on the regulatory mechanism of *HcSWEET* genes in response to abiotic stress in detail.

## Materials and methods

### Identification and characterization of the *HcSWEET* genes in night lily

The whole genome sequences of night lily were downloaded from NCBI(https://www.ncbi.nlm.nih.gov/assembly/GCA_017893485.1_ASM1789348v1_genomic).To identify all members of *HcSWEET* genes, the protein sequence of SWEETs in *Hemerocallis citrina*, *Zea mays* and *Vitis vinifera* also were obtained from National Center for Biotechnology Information (NCBI), *Arabidopsis thaliana* from the Arabidopsis Information Resource (TAIR) [76], *Oryza sativa* from Ensembl Genome database [77], and *Hemerocallis fulva* from reported data previously [41], respectively. Functional genes with incomplete domains were supplemented and screened by searching conserved domains (CD-Search) [78]. Molecular weight (MV) and theoretical isoelectric points (PI) were detected using TBtools [79]. Transmembrane topology is predicted by Deep TMHMM Server v1.0.24. Subcellular localization analysis was preliminarily predicted via WoLF PSORT software online [80].

### Secondary and tertiary structure predictions

The secondary structures of 19 HcSWEET proteins were predicted by the SOPMA tool online. The three-dimensional (3D) structures of these HcSWEET proteins were constructed using homology modeling. All 19 protein sequences were aligned with all other proteins of the Protein Data Bank (PDB), and the optimum models were finally built with a standard of relatively high GMQE and seq identity values by Swiss-Model [81].

### Phylogenetic tree construction, gene structure analysis, and prediction of conserved motifs and domains

Multiple Alignment Trimming in TBtools was used to carry out protein sequence alignments in night lily and other four plants. Based on the primary results of sequence alignment, trimAL Wrapper proceeded to have a shear to remove non-conserved regions as soon as possible. After shears were completed, a phylogenetic tree was constructed by One Step Build an ML Tree with 5000 bootstrap replicates. The visualization of gene structure was performed by Visualize Gene Structure of TBtools. MEME online and NCBI conserved domain database were respectively used to predict the conserved motifs and domains of the HcSWEETs.

### Chromosomal location, collinearity, and cis-acting element (CREs) analysis

The genome annotation file in GFF format of *HcSWEETs* was obtained from the NCBI database. Both *Arabidopsis* and rice genomes were downloaded from the Ensembl Plants database. Intra-species and intre-species collinear analysis about night lily were constructed via MCScanX and TBtools software [79, 82]. A 2000 bp sequence in the promoter region was extracted utilizing TBtools from the genome. The online PlantCARE database was employed to predict the CREs of interest [83].

### Plant materials and stress treatments

Night lily F1 hybrid population 116 (Dongzhuang Huanghua as female parent and Chonglihua as male parent) was used as treated plant material in this study, which was grown in the experimental base of Shanxi Agricultural University in Taigu under natural conditions and managed in the same regular agricultural practice. Population 116 showed moderate resistance to abiotic stress compared with the daylily cultivar ‘Golden Doll’. The experiments were conducted in 2023, and plants used for experimental treatments were from tissue cultivation. These seedlings through domestication were cultured in the same conditions for one month, after which drought and salt treatment were carried out. Drought and salt stress were respectively simulated using PEG6000 (20%) and NaCl (250 mM). Drought stresses were designed into 5 groups (0 h, 24 h, 48 h, 72 h, 108 h), and salt treatments were divided into 5 groups (0 h, 24 h, 48 h, 72 h, 96 h). Roots of treated plants were collected and immediately stored at -80 °C for follow-up experiments.

### Comparative transcriptome analysis

To analyze tissue-specific expression patterns, different tissues including tender root, mature root, bud, tender leaf, mature leaf, tender scape, and mature scape were collected. Total RNA of different tissues and treated roots was isolated using the TIANGEN RNAsimple Total RNA Kit, and RNA quality was evaluated by 1.5% (w/v) agarose gel. Samples with three biological replicates were selected and sent to BMK Biotechnology Company to conduct an RNA-seq assay. Before analyzing the RNA-Seq data, quality control checks were performed through trimmomatic software [84], followed by mapping high-quality reads to the *H. citrina* reference genome using HISAT2 [85]. Differentially expressed genes (DEGs) were identified and modelled using DESeq2 tools [86], and filtered with standreds of Fold Change ≥ 2 and False Discovery Rate < 0.05. The average value of FPKM (reads per kilobase per million reads) was used to measure gene expression levels in the study. The heat map was drawn with log_2_FPKM values to show different expression levels intuitively.

### RT-qPCR analysis

Based on RNA-seq results, an RT-qPCR assay was performed for further verification of *HcSWEETs* expression levels with *HcACTIN* as the standard. Real-time quantitative PCR amplification was performed by PerfectStart^®^ Green SuperMix (TransGen, China). RT-qPCR procedure with triplicate was completed using a Real-Time PCR System (ABI QuantStudio 5, USA), and lg2^−ΔΔCt^ values were taken as a measure of relative expression levels. SPSS 20 software was used to calculate statistical data and different letters represented significant differences between different treatments in accordance with Duncan’s multiple test. All gene-specific primers were given in Supplementary Table S2.

### Construction of *HcSWEET4a* transient expression vector and subcellular localization

The first-strand cDNA of night lily was synthesized using TransScript^®^ One-Step gDNA Removal and cDNA Synthesis SuperMix (TransGen, China). Night lily WT cDNA was used as template to amplify the coding sequence without stop codon of *HcSWEET4a* using primers containing the XbaI/KpnI restriction sites. PCR product purified was sub-cloned into enzyme-digested pSuper1300-GFP vector by the homologous recombination method using Monclone Single Assembly Cloning Mix (Monad, China). The recombinant pSuper1300-HcSWEET4a-GFP was transformed into *Agrobacterium tumefaciens* strain GV3101. Tobacco leaf cells were transiently injected by the cultures. After 16–24 h, fluorescence images were captured under a fluorescence microscope (Leica STELLARIS 5, Germany). Relative primers were listed in Supplementary Table S2.

### Overexpression vector construction and transformation of *HcSWEET4* in watermelon

The PCR product with the KpnI and BamHI restriction sites was obtained by using binary pSuper1300-HcSWEET4a-GFP as template, then was cloned into 1305.4-GFP vector and sequenced. Constructed 1305.4-HcSWEET4a-GFP vector was transformed into watermelon YL using an optimized genetic transformation system [87]. Transgenic watermelon plants were screened by GFP fluorescence observation and PCR analysis. WT and positive plants were transplanted to the same growing conditions to cultivate. The salt tolerance of transgenic plants was analyzed with WT watermelon plants as the negative control. Primers referred to in the experiment were given in Supplementary Table S2.

### Electronic supplementary material

Below is the link to the electronic supplementary material.

Supplementary Material 1

Supplementary Material 2

Supplementary Material 3

## Data Availability

The data that support results are included in this manuscript and supplementary information files. Other relevant materials also are available from corresponding authors upon reasonable request.

## Declarations

### Voucher specimen

The authors declare that there are no voucher specimen included in the study.
