# Supplementary material for: Genome-wide analysis of the SWEET gene family in Hemerocallis citrina and functional characterization of HcSWEET4a in response to salt stress
Source: BMC Plant Biol. 2024 Jul 11;24:661. doi: 10.1186/s12870-024-05376-y (PMC11238388; doi:10.1186/s12870-024-05376-y)
Supplement: Supplementary file 1 — Supplementary Material 1 [file 12870_2024_5376_MOESM1_ESM.docx]

**Supplementary Information 1** Protein sequences used for phylogenetic analysis among different species

*Hemerocallis citrina*

> HcSWEET1a

METVLHFVFGMFGNITALFLFFSPIVTFRRIIKNRSTEEFSGVPYNMTMLNCLLSAWYGLPFVSPNNLLVWTINGTGVLIEAIYVLIFLIFAPKKVRSRMMGLLALVSSVFISVALISVLALHGQSRKVFCGLAATIFSICMYGSPLSIMRLVIRTKSVEYMPFFLSLFVFLCGTSWFIYGLLGHDVFIAVPNGCGSALGLVQLILYAIYRNHKGNKSSDTNGESLQMTDVKPTDNYAKNVEQV

>HcSWEET1b

MENVLHFVFGIFGNVTALFLFFSPMVTFRRIIKNRSTEEFSGVPYNMTMLNCLLSAWYGLPFVSPNNLLVWTINGTGALIEAIYVLIFLIFAPKKVRGRMMGLLALVSSVFISVALISVLALHGQTRKFFCGFAATIFSICMYGSPLSIMRLVIRTKSVEYMPFFLSLFVFLCGTSWFIYGLLGRDVFIAVPNGCGSALGLVQLIIYAIYRNHKGDKSTDTNGESLQMTDVKPTDNYEKNVKPTNNYEKNVEEV

> HcSWEET2

MGPSSFYELCTYAAGIAGNLFAFVLFVSPLPTFRRIIRNRSTEQFSGLPYVYSLLNCLICFWYGLPFVSRGVILMCTVNSVGAAFQLVYITVYILFADSSGKVKISGLLIAVFGAFALIMYITLQYFDHGMRKMVVGYLSIATLISMFASPLSVINLVIRTKSVEFMPFYLSLATFLMSISFFAYGMLLQDFFVYLPNGIGSILGAIQLLLYAYYSKNVSRDTRLPLLQ

> HcSWEET3

MGTSLRFPVGILGNAASLFLYTAPILTFARVIRKRSTEEFSCIPYIIALLNCLLYTWFGLPVVSKGWENFTVATINGLGILLEISFILIYIWFALAKRKKFAISMLVAVIVVFGMTAFVSSVVLHDHPHRKVFVGSVGLVASVAMYSSPLVAMRLVIKTKSVEFMPFYLSFFSFLASSLWMLYGLLGQELFIAAPNFLGTPVGILQLILYCMYKKKKGGHQEPKNIDVENNGEKQLNS

> HcSWEET4a

MVSADTIRTCVGILGNLIALALFLSPVPTFCRIWKKGSVEQFSAVPYLATLLNCMLWVVYGLPLVHPHSTLVLTINGSGLIIELSYVLLFIIFSHGSNRLRVVIILVSEIVFVAFAGLLVIIFTHTFAMRSLIIGILCVFFGTMMYAAPLSVMKLVIQTKSVEFMPLFLSLASFFNGLCWTTYALIHFDLYITIPNGLGVLFAMAQLVLHIMYYKTTKEQMEARKRKTETALSEVVVFGDNNNKVGKGSQNGVATHP

> HcSWEET4b

MVNTETIRTVVGIIGNVISLGLFLSPAPTFVGIIKKKDVEQFSPVPYLATLLNCMLWVLYGLPIVHPDSTLVITINGAGVVIELIYIAIFVIFSDGKKRLNVFLIFIGEVIFTFTFGVLVIELLHTTTRRSTLVGILCVIFCIMMYVAPLSVMRMVIKTKSVEYMPLFISVASFCNGACWTVYSLLKFDLNILIPNGIGLVFSVVQLILYAVFYKSTQRLLEARKKAEVGMTGMGQADKLSDVV

> HcSWEET4c

MVNTETIRTIVGVVGNVISFGLFLSPVPTFVGIVKRKDVEQFSPVPYLATLLNCMLWVLYGLPIVHPDSTLVLTINGAGVVIELIYIAVFITFCDGKKRFNVILIFLGEIIFTVTFGVLVIELLHTTTRRSTVVGILCVIFCIMMYVAPLSVMRMVIKTKSVEYMPLFLSVASFCNGTCWTVYSLLKFDLNILIPNGIGLLFSVVQLILYAVFYKSTQQILEARKKAEVGMTGMGQADKISNAV

> HcSWEET5

MVSADTIRTCVGILGNVIALVLFLSPVPTFYRIWKKGSVEQFSAVPYLATLLNCMLWVVYGLPLVHPHSTLVLTINGSGLVIELSYVLLFIIYSHGSNRLRVVAILVSEIIFVALSGLLVIIFTHTIAMRSLIIGILCVFFGTMMYAAPLSVMKLVIQTKSVEFMPLFLSLASFFNGLCWTTYALIRFDLYITIPNGLGVLFAMAQLVLHIIYYKTTKEQMEARKRKAETGLSEVVVFGDNNNKVGKGSQNGVP

> HcSWEET6

MVSADTIRTVVGILGNVISLILFLSPAPTFIRIWKKGSVEQFSPVPYLATLLNCLLWVVYGLPLVHPHSTLVLTINGSGVAIELFYVLLFIIYSQGSKRLRVLIILVSEIASVMLVGVLVIVFAHTLAMRSLIIGILCVFFGTMMYAAPLSVMKLVIQTKSVEFMPLFLSLASFFNGLSWTAYALIRFDLFVTIPNALGVIFAVAQLVLHIMYYKSTKEQMEARKRKAETGLSEVVVHADSNTKVNDAPN

> HcSWEET11

MAGLSFDHPWVLIFGLLGNILSGMVYLSPVPTFRRICRKKSTEGFQSFPYCVALFSAMQLIYYAIIKTNTYLLITINTAGCVIESAYIIIYLIYAPKKARINTVKTMLLLNVGLFSAIVLISFLCFKGADRVKVVGWINMTFSVSVFAAPLSIIRLVIRTKSVEFMPFFLSFFLTMSAIAWFFYGLLTRDIYVAFPNILGFTFGCIQMILYIVYKDAKKGKEDIEGKLPEEVLDDTAKPSPMGVEVSKAETAEDNTSRVDNASQV

> HcSWEET13a

MAGLSLDHPWAFAFGLLGNLISFMVLLAPIAMLWIYYAFVKTNEYLLITINTFGCFIETLYIVMYLTYAPRKAKVNTAKIFLLLNVGLFSLIVLATIFLSKGANRQKLLGWICVGFAVSVFAAPLSIIKQVIKTKSVEYMPFSLSFFLTLSAVVWFAYGLLIKDIYVAIPNILGFVFGIIQMVLYIIYNDVKVLKEELKIGESTEGKMGEITVEVEIEKGDTTVKGGKKMSMEKAKMGLKDGVEMCQV

> HcSWEET13b

MAGLSLDHPWAFAFGILGNLISFMVCLAPIPTFYRIYKSKSTQGFQSVPYVVALFSAMLWIYYALIKTNEYLLITINSVGCVIETIYIIIFITYASKKIKIHTVKLMLLLNVGLFASIVLSTLLLTKGPTRVTVVGWMCVGFAVSVFAAPLSIIRQVMRTRSVEFMPFSLSFSLTLSAVAWFSYGLLTKDIYVAIPNILGFSFGVVQMVLYIIYKDAKELVKDDKVDETEHVVISIAKLNESRVAPQVSASEQELVQAAEENNKEEEEEGKEMLEVEGQNAIEMSSPV

> HcSWEET13c

MAGLSLDHPWAFAFGILGNLISFLVYLAPIPTFYRIYKSKSTQGFQSVPYVVALFSAMLWIYYAFIKTNEYLLITINSVGCVIETVYIIMFITYASKKIKIHTVKLMLLLNVGLFSSIVLSTLLLAKGPKRVTVLGWMCVGFAVSVFAAPLSIIRQVIRTRSVEFMPFSLSFSLTLSAVVWFSYGLLTKDIYVAIPNILGFSFGVVQMILYIIYKDAKELVKDNKLDEKEQVVISIPRLNESRVAPQGDQELVKAGKENNKEEEKEMVEVEGQNGIEMSPV

> HcSWEET13d

MVYLAPIPTFYRIYRKKSTEGFQSLPYVVALFSAMLWIYYAFIKTDEYLLITINTVGCVIETIYIIMFLAYAPKKVKIHTVKMILLLNVGLFSLIVLSTLLLSKGADRVKVLGWMCVGFAVGVFAAPLSIIRQVIRTKSVEFMPFSLSFSLTLSAIVWFSYGLLTKDIYVAIPNILGFSFGTVQMVLYLIYKDAKDLMINEDKLPNTKVISQNNVNTEDTKLDEEKKGYHEDKKDMVEGKDEIELSPV

> HcSWEET14a

MAGLSLDHPWAFTFGLLGNLISFMVYLAPIPTFHRIYKKKSTEGFQSVPYVVALFSAMLWIYYAFIKSNEYLLITINSLGIVIETVYIIMFIAYAPKKAKVYTAKIIMGLNVGLFSLIVLSTLLLTKGAHRQKLLGWMCVGFAVSVFAAPLSIIRQVIRTKSVEFMPFSLSFFLTLSAIVWFSYGLLIKDIYVAIPNILGFSFGVIQMILYIIYKDVKVLKEELKLPEDLATMAKLGEIMIEAQGIAVEIEVKEEKMKRVKEEEKKITGIEQAIKGEN

> HcSWEET14b

MAGLSLDHPWAFTFGLLGNLISFMVYLAPIPTFHRIYKKKSTEGFQSVPYVVALFSAMLWIYYAFIKSNEYLLITINSLGIVIETVYIIMFIAYAPKKAKVYTAKIIMGLNVGLFSLIVLSTLLLTRGANRQKLLGWMCVGFAVSVFAAPLSIIRQVIRTKSVEFMPFSLSFFLTLSAIVWFSYGLLIKDIYVAIPNILGFSFGVIQMILYVIFKDVKVLKEELKLPEDLATMAKLGEIMIEAEGIAVEIEVKEEKMKKVKEEKKITGIEQAIKGEKDEVEMCQV

> HcSWEET14c

MAGLSLHHPWAFTFGLLGNVISFMVYLAPIPTFYRIYKKKSTEGFQSVPYVVALFSAMLWIYYAFIKTNEYLLITINSFGIVIETVYTVMFLAYAPKKAKVYTTKLIMGLNVGLFSLIVLSTLFLTKGAKRQKLLGWICVGFAASVFAAPLSIIRQVIRTKSVEFMPFWLSFFLALSAMVWFSYGLLIKDIYVAIPNILGFSFGVVQMVLYIIYKDVKVLKEELKLPEDLTTMARLGKNVIEAQEITAEIEVKGDKKKVKEEEKKIIGMEKAIKGEKDDVEMCQV

> HcSWEET15

MCSMAEEHHWAFAFGILGNIISFMVYLAPLPTFLRIYKKKSTQGFQSIPYVVALFSASLWIYYAILKSTNTFLLITINAAGCVIEAGYIIFYLIYAPRNARIYTAKLLLLLNVGLFGLIILLTLLLSTGSGRVVTLGWICVGFSTSVFVAPLSIIRLVIQTKSVEFMPFSLSFSLTLSAVVWFFYGFLSHDIYIALPNVLGFIFGVIQMLLYAMYMSENKPKIVEQHHAMHEQIITIAELGVVLGLEVDLVDTATDASKENIGGQGNECECECELTNIQGQEGINEMIL

> HcSWEET16

MAAPSLIVGIVGNVISILVFASPITTFWRIVKKKSTMNYEGLPYVTTLLCTSMWTYYGLHKPGGLLIVTVNGAGSVMQSVYVVLFLIYAQTSTRIRVGRLVGILNVGVFGMVILVTSLALHGNLRLLVVGCMCAGLTVGMYAAPMAVMRLVVQTRSVEYMPFSLSFFLFLNGGVWGAYAFLVKDFFIGIPNVIGFVLGTAQLILYATYRKKSPVAKEVDLEMGRMDQIAEQKLKMASNAQDKNHLHRGASLPMKRSVSRQRSLTKIVKSLSLPPYENPDWSLDDLDNHSEVEHPKQILG

*Hemerocallis fulva*

>HfSWEET1a

METVLHFVFGMFGNITALFLFFSPIVTFRRIIKNRSTEEFSGVPYNMTMLNCLLSAWYGLPFVSPNNLLVWTINGTGVLIEAIYVLIFLIFAPKKVRSRMMGLLALVSSVFISVALISVLALHGQSRKVFCGLAATIFSICMYGSPLSIMRLVIRTKSVEYMPFFLSLFVFLCGTSWFIYGLLGHDVFIAVPNGCGSALGLVQLILYAIYRNHKGNKSSDTNGESLQMTDAKPTDNYAKNVEQV

>HfSWEET1b

MENVLHFVFGIFGNVTALFLFFSPMVTFRRIIKNRSTEEFSGVPYNMTMLNCLLSAWYGLPFVSPNNLLVWTINGTGALIEAIYVLIFLIFAPKKVRGRMMGLLALVSSVFISVALISVLALHGQTRKFCCGFAATIFSICMYGSPLSIMRLVIRTKSVEYMPFFLSLFVFLCGTSWFIYGLLGRDVFIAVPNGCGSALGLVQLIIYAIYRNHKGDKSTDTNGESLQMTDVKPTDNYEKNVKPTNYEKNVEEV

>HfSWEET2a

MSSSELSSFYTICSYSAGIAGNLFALVLFIAPMHTFGRIIRNKSTEQFSGLPYIYALLNCLICFWYGLPFVSHGVILVSTVNSIGACFQLAYVIIFIVFADTRGKLKMSGLLVGVLVVFALITYCSLEFFDHSMRQLFIGYLSIASLISMFASPLSVIRLVVRTRSVEFMPFYLSLATFLMSIAFFAYGMLLEDFFIYLPNGIGSILGVIQLLLYVYFNKSLRGDSRMALLV

>HfSWEET3b

MGSSLRFPVGILGNAASLFLYTAPILTFARVIRKGSTEEFSCIPYIIALLNCLLYTWFGLPVVSKGWENFTVATINGLGILLEISFILIYIWFASAKRKKLVIPMVVAVIVVFGITAFVSSAVFHDHPHRKVFVGSVGLVASVAMYGSPLVAMRLVIKTKSVEFMPFYLSFFSFLASSLWMLYGLLGQELFIAAPNFLGTPMGILQLILYCMYKNKKGAHEEQRNIDIEKNGEKLNS

>HfSWEET4a

MVSADTIRTCVGILGNVIALALFLSPVPTFYRIWKKGSVEQFSAVPYLATLLNCMLWVVYGLPLVHPHSTLVLTINGSGLVIELSYVLLFIIYSHGSNRLRVVAILVSEIVFVALSGLLVIIFTHTIAMRSLIIGILCVFFGTMMYAAPLSVMKLVIQTKSVEFMPLFLSLASFFNGLCWTTYALIRFDLYITIPNGLGVLFAMAQLVLHIIYYKTTKEQMEARKRKAETGLSEVVVFGDNNNKVGKGSQNGVANHP

>HfSWEET4b

MVNTETIRTVVGIIGNVISLGLFLSPAPTFVGIIKKKDVEQFSPVPYLATLLNCMLWVLYGLPIVHPDSTLVITINGAGVVIELIYIAIFVIFSDGKKRLNVFLIFIGEVIFTFTFGVLVIELLHTTTRRSTLVGILCVIFCIMMYVAPLSVMRMVIKTKSVEYMPLFISVASFCNGACWTVYSLLKFDLNILIPNGIGLVFSVVQLILYAVFYKSTQRLLEARKKAEVGMTGMGQADKLSDAV

>HfSWEET4c

MVNTETIRTIVGVVGNVISFGLFLSPVPTFVGIVKRKDVEQFSPVPYLATLLNCMLWVLYGLPIVHPDSTLVLTINGAGVVIELIYIAVFVTFCDGKKRFNVILIFLGEIIFTVTFGVLVIELLHTTTRRSTVVGILCVIFCIMMYVAPLSVMRMVIKTKSVEYMPLFLSVASFCNGTCWTVYSLLKFDLNILIPNAIGLLFSVVQLILYAVFYKSTQQILEARKKAEVGMTGMGQADKISNAV

>HfSWEET5

MVNAEVIRSGVGIVGNAISLGLFLSPLPTFVQIIKQKAVEQFSPVPYLATLLNCLLWFFYGLPIVHLNGMLILTINGAGLLFETVYLSIFLTFAPPNQRLKVIGIIIGELVFVGAVAAVVLNVAHTIPKREMMVGILCVIFGTCMYASPLSVMKLVIETKSVKYMPFTLSLVSFVNGLCWTSYALLKFDLYVLIPNGLGTMLGLIQLIIYAIYFKSTQTTGDLPTKSEKSVEMQGEL

>HfSWEET6a

MVGVDAIRNIVGIIGNVISFGLFLSPVPTFVTIINRKAVEEFSPIPYLATLLNCMLWVFYGIPLVHPHSILVVTINAIGLILESIYLIIFFIYSPPKLRLKVVKILAGELVFMAAVITGVIVGAHTHERRSLVVGILCVIFGTMMYASPLSIMKLVIQTKSVEYMPFFLSLVAFLNGVCWTTYALLRFDIFMTIPNGLGALLGLAQLILYFCYYNSTPKRRSKMEVELPTVNPAASS

>HfSWEET6b

MVSADTARTIVGIIGNVISFGLFLSPLPTFVKIVKKKAVEDFSAIPYLATLLNCMLWVFYGLPVTHPHSTLVYTINGIGLVIEGAYLITYFIYAPPNGRCKVLKILAGELVFMAAVILGVLLGAHTHHKRSLVVGILCIIFGTCMYASPLSIMKLVIQTKSVEYMPFFLSLAGFLNGVCWTAYALIKFDINLLIPNGLGALFGLAQLILYFCYYSSTPKKTSKPEFELPTNLTDNAMSEVEDGICSANGSLRANGQRRAARSFQLLWPVASLSLISTNM

>HfSWEET7

MVNIDAIRTTVGIIGNVISFGLFLSPVPTFIAIIKKKAVEQFSPIPYVCTLLNCFLWCLYGLPIVHPDATLVITINGVGIVIELIYISIFVIYAPGSKKVQVLLTVLAEAVFVVVFGILVIRFFPTTARRSTVVGILCIVFNIIMYVSPLSVMKLVIQTKSVEFMPFPLSFASFCNGACWTIYSVLKFDVNIFLPNGIGTLFSVVQLVLIAIFWSNTQKIIAARKNGGGGDVAMTS

>HfSWEET12a

MLVITIQDPWLFGVGLLGNITSFLVVIAPVPTFYRICKKKSTESFQSIPYAVAVFSATLWLYYAALTSDLLLLTINTAAVFIEASYLAIYLIYASKKARAFTMKLIFLLDVGFYGSLVLLTLLFLQGKKRINLVGMICAAFAVSVFVAPLSIIKLVIRTKSVEYMPFTLSFFLTLSALAWFCYGLLLKDSYIALPNVVGFLFGMVQMILYFIYMNSKREEPQPNNQVLDNNPITRSTTSDIEIEVIDCKKTSSESPARVEA

>HfSWEET13a

MAGLSLDHPWAFAFGLLGNLISFMVLLAPIPTFYRIYKKKSTEGFQSVPYVVALFSAMLWIYYAFVKTNEYLLITINTFGCFIETLYIVMYLTYAPRKAKVNTAKIFLLLNVGLFSLIVLATIFLSKGANRQKLLGWICVGFAVSVFAAPLSIIKQVIKTKSVEYMPFSLSFFLTLSAVVWFAYGLLIKDIYVAIPNILGFVFGIIQMVLYIIYNDVKVLKEELKIGESTEGKMGEITVEVEIEKGDTTVKGGKKMSMEKAKMGLKDGVEMCQV

>HfSWEET13b

MAGLSLDHPWAFAFGILGNLISFMVCLAPIPTFYRIYKSKSTQGFQSVPYVVALFSAMLWIYYALIKTNEYLLITINSVGCVIETIYIIIFITYASKKIKIHTVKLMLLLNVGLFASIVLSTLLLTKGPKRVTVVGWMCVGFAVSVFAAPLSIIRQVMRTRSVEFMPFSLSFSLTLSAVAWFSYGLLTKDIYVAIPNILGFSFGVVQMVLYIIYKDAKELVKDDKVDETEHVVISIAKLNESRVAPKVSASEQELVQPAEENNKEAEEEEEGKEMLEVEGQNAIEMNPV

>HfSWEET14a

MAGLSLDHPWAFTFGLLGNLISFMVYLAPIPTFHRIYKKKSTEGFQSVPYVVALFSAMLWIYYAFIKSNEYLLITINSLGIVIETVYIIMFIVYAPKKAKVYTAKIIMGLNVGLFSLIVLSTLLLTKGAHRQKLLGWMCVGFAVSVFAAPLSIIRQVIRTKSVEFMPFSLSFFLTLSAIVWFSYGLLIKDIYVAIPNILGFSFGVIQMILYIIYKDVKVLKEELKLPEDLATMAKLGEIMIEEQGIAVEIEVKEEKMKRVKEEEKKITGIEQAIKGEKDEVEMCQV

>HfSWEET14b

MAGLSLDHPWAFTFGLLGNLISFMVYLAPIPTFHRIYKKKSTEGFQSVPYVVALFSAMLWIYYAFIKSNEYLLITINSLGIVIETVYIIMFIAYAPKKAKVYTAKIIMGLNVGLFSLIVLSTLLLTRGANRQKLLGWMCVGFAVSVFAAPLSIIRQVIRTKSVEFMPFSLSFFLTLSAIVWFSYGILIKDIYVAIPNILGFSFGVIQMILYIIYKDVKVLKEELKLPEDLATMAKLGEIMIEAEGIAVEIEVKEEKMKKVKEEEKKITGIEQAIKGEKDEAEMCQV

>HfSWEET15

MCSMAEEHHWAFAFGILGNIISFMVYLAPLPTFLRIYKKKSTQGFQSIPYVVALFSASLWIYYALLKSTNTFLLITINAAGCVIEAGYIIFYLIYAPRNARIYTAKLLLLLNVGLFGLIILLTLLLSTGSGRVVTLGWICVGFSTSVFVAPLSIMRLVIQTKSVEFMPFSLSFSLTLSAVVWFFYGFLSHDIYIALPNVLGFIFGVIQMLLYAMYMSENKPKIVEQHHAMHEQIITIAELGVVLGLEVDLVDTATDASKENIGGQGNECECECELTNIQGQEGINEMIP

>HfSWEET16

MAAPSLIVGIVGNVISILVFASPVTTFWRIVKKKSTMNYEGLPYVTTLLCTSMWTYYGLHKPGGLLIVTVNGAGSVMQSVYVVLFLIYAQTSTRIRVGRLVGILNVGVFGMVILVTILALHGNLRLLVVGCMCAGLTVGMYAAPMAVMRLVVQTRSVEYMPFSLSFFLFLNGGVWGAYAFLVKDFFIGIPNVIGFVLGTAQLILYATYRKKSPVAKEVDLEMGRMDQIAEQKLKMASNAQDKNHLHRGASLPMKRSVSRQRSLTKIVKSLSLPPYENPDWSLDDLDNHSEVEYPKQILG

>HfSWEET17

MESLLFIIGVVGNVTSLLIFLSPMTTFWRIMKNKSTEDFEPVPYVVTLLGCSLWVYYGITKPGEYLVATVNGVGIIFEAIYVVIFLIYATPPLRVKTSIMVVVLDGAILGAVVLATQLVAEEGLRIMIIGVICACLNIFMYGSPLTIMKTVITTRSVEYMPFFLSFFLFVNGGIWTVYAIIDSDFFLGIPNGIGFIFGAIQLTLYMIYMNTKVSKQYLEESPRQRLLNEQEEDVEEGRPT

*Oryza sativa*

>OsSWEET1b

MEDLAKFLFGVSGNVIALFLFLSPVPTFWRIIRRKSTEDFSGVPYNMTLINCLLSAWYGLPFVSPNNILVSTINGAGAVIETAYVVVFLVFASTHKTRLRTLGLAAAVASVFAAVALVSLLALHGQHRKLLCGVAATVCSICMYASPLSIMRLVIKTKSVEYMPFLMSLAVFLCGTSWFIYGLLGRDPFVTIPNGCGSFLGAVQLVLYAIYRNNKGAGGGSGGKQAGDDDVEMAEGRNNKVADGGAADDDSTAGGKAGTEV

>OsSWEET2a

MMNALGLSVAATSTGSPFHDVCCYGAGIAGNIFALVLFISPLPTFKRIVRNGSTEQFSAMPYIYSLLNCLICLWYGLPFVSYGVVLVATVNSIGALFQLAYTATFIAFADAKNRVKVSSLLVMVFGVFALIVYVSLALFDHQTRQLFVGYLSVASLIFMFASPLSIINLVIRTKSVEYMPFYLSLSMFLMSVSFFAYGVLLHDFFIYIPNGIGTVLGVIQLVLYGYFRKGSREDSLPLLVTHT

>OsSWEET2b

MDSLYDISCFAAGLAGNIFALALFLSPVTTFKRILKAKSTERFDGLPYLFSLLNCLICLWYGLPWVADGRLLVATVNGIGAVFQLAYICLFIFYADSRKTRMKIIGLLVLVVCGFALVSHASVFFFDQPLRQQFVGAVSMASLISMFASPLAVMGVVIRSESVEFMPFYLSLSTFLMSASFALYGLLLRDFFIYFPNGLGLILGAMQLALYAYYSRKWRGQDSSAPLLLA

>OsSWEET3a

MFPDIRFIVGIIGSVACMLLYSAPILTFKRVIKKASVEEFSCIPYILALFSCLTYSWYGFPVVSYGWENMTVCSISSLGVLFEGTFISIYVWFAPRGKKKQVMLMASLILAVFCMTVFFSSFSIHNHHIRKVFVGSVGLVSSISMYGSPLVAMKQVIRTKSVEFMPFYLSLFTLFTSLTWMAYGVIGRDPFIATPNCIGSIMGILQLVVYCIYSKCKEAPKVLHDIEQANVVKIPTSHVDTKGHNP

>OsSWEET3b

MVSNTIRVAVGILGNAASMLLYAAPILTFRRVIKKGSVEEFSCVPYILALFNCLLYTWYGLPVVSSGWENSTVSSINGLGILLEIAFISIYTWFAPRERKKFVLRMVLPVLAFFALTAIFSSFLFHTHGLRKVFVGSIGLVASISMYSSPMVAAKQVITTKSVEFMPFYLSLFSFLSSALWMIYGLLGKDLFIAVSTYRSPNFIGCPMGILQLVLYCIYRKSHKEAEKLHDIDQENGLKVVTTHEKITGREPEAQRD

>OsSWEET4

MVSPDTIRTAIGVVGNGTALVLFLSPVPTFIRIWKKGSVEQYSAVPYVATLLNCMMWVLYGLPAVHPHSMLVITINGTGMAIELTYIALFLAFSLGAVRRRVLLLLAAEVAFVAAVAALVLNLAHTHERRSMIVGILCVLFGTGMYAAPLSVMKMVIQTKSVEYMPLFLSLASLVNGICWTAYALIRFDLYITIPNGLGVMFAVAQLILYAIYYKSTQQIIEARKRKEADHVAMTDVVVDSAKNNPSSGAAAAAANGRY

>OsSWEET5

MVMNPDAVRNVVGIIGNLISFGLFLSPLPTFVTIVKKKDVEEFVPDPYLATFLNCALWVFYGLPFIHPNSILVVTINGTGLLIEIAYLAIYFAYAPKPKRCRMLGVLTVELVFLAAVAAGVLLGAHTYDKRSLIVGTLCVFFGTLMYAAPLTIMKQVIATKSVEYMPFTLSLVSFINGICWTIYAFIRFDILITIPNGMGTLLGAAQLILYFCYYDGSTAKNKGALELPKDGDSSAV

>OsSWEET6b

MISPDAARNVVGIIGNVISFGLFLSPVPTFWRICKRKDVEQFKADPYLATLLNCMLWVFYGIPIVHPNSILVVTINGIGLIVEGTYLFIFFLYSPNKKRLRMLAVLGVELVFMLAVILGVLLSAHTHKKRSMIVGILCVFFGSIMYFSPLTIMGKVIKTKSVEYMPFFLSLVCFLNGVCWTAYALIRFDIYVTIPNGLGAIFGAIQLILYACYYRTTPKKTKAAKDVEMPSVISGPGAAATASGGSVVSVTVER

>OsSWEET7a

MVSPDMIRNVVGIVGNVISFGLFLSPVPTFWQIIKNKNKNKKKMEVVLAAEALFMVSPDMIRNVVGIVGNVISFGLFLSPVPTFWQIIKNKNKNKKKMEVVLAAEALFMAAVALGVLLGVHTHQRRSLIVGILCVIFDTIMYSSPLTVMSQVVKTKSVEYMPLLLSVVSFLNGLYWTSYTLIRFDIFITIPNGLGVLFAAVQLILYVIYYRTTPKKQNKNLELPTVTPVAKDTSVGPISKDNDLNGSTASHVTIDITIQP

>OsSWEET7b

MVSPDLIRNMVGIVGNIISFGLFLSPVPTFYRIIKNKDVQDFKADPYLATLLNCMLWVFYGLPIVHPNSILVVTINGIGLVIEAVYLTIFFLFSDKKNKKKMGVVLATEALFMAAVVLGVLLGAHTHQRRSLIVGILCVIFGTIMYSSPLTIMSQVVKTKSVEYMPLLLSVVSFLNGLCWTSYALIRLDIFITIPNGLGVLFALMQLILYAIYYRTIPKKQDKNLELPTVAPVAKDTSIVTPVSKDDDVDGGNASHVTINITIEL

>OsSWEET7c

MVSPDLIRNVVGIVGNVISFGLFLSPVPIFWRIIKNKNVQNFKADPILVVTINGISLVIEAVYLTIFFLFSDKKNKKKMGVVLATEALFMAAVAVGVLLGAHTHQRRSLIVGILCVIFGTIMYSSPLTIMVVKTKSVEYMPLLLSVVSFLNGLCWTLYALIRFDIFITIPNGLGVLFAIMQLILYAIYYRTTPKKQDKNLELPTVAPIAKDTSIVAPVSNDDDVNGSTASHATINITIEP

>OsSWEET7d

MVPDLIRNVVGIVGNVISFGLFLSPVPTFWRIIKNKDVRDFKADQYLATLLNCMLWVFYGLPIVHPNSILVVTINGIGLVIEAVYLTIFFLFSDKKNKKKMGVVLATEALFMAAVALGVLLDAHTHQRRSLIVGILCVIFGTIMYSSPLTIMSQVVKTKSVEYMPLLLSVVSFLNGLCWTSYALIRFDIFITIPNGLGVLFALMQLILYAIYYRTTPKKPSTTGPHPRSRIRTSSYQPSPPSPRAPASSPLSARTTTSMAAMSPSISRLSHKLA

>OsSWEET7e

MVSPDLIRNVVGIVGNAISFGLFLSPVLTFWRIIKEKDMKYFKADPYLATLLNCMLWVFYGLPIVHPNSILVVTINGIGLVIEAVYLTIFFLFSNKKNKKMGVVLATEALFMAAVALGVLLGAHTHQRRSLIVGILCVIFGTIMYSSPLTIMSQVVKTKSVEYMPLLLSVVSFLNGLCWTSYALIRFDIFITIPNGLGVLFTLMQLILLSW

>OsSWEET11

MAGGFLSMANPAVTLSGVAGNIISFLVFLAPVATFLQVYKKKSTGGYSSVPYVVALFSSVLWIFYALVKTNSRPLLTINAFGCGVEAAYIVLYLVYAPRRARLRTLAFFLLLDVAAFALIVVTTLYLVPKPHQVKFLGSVCLAFSMAVFVAPLSIIFKVIKTKSVEFMPIGLSVCLTLSAVAWFCYGLFTKDPYVMYPNVGGFFFSCVQMGLYFWYRKPRNTAVLPTTSDSMSPISAAAAATQRVIELPAGTHAFTILSVSPIPILGVHKVEVVAAEQAADGVAAAAAADKELLQNKPEVIEITAAV

>OsSWEET12

MVQALVFAVGIVGNILSFLVILAPVPTFYRVYKKKSTESFQSVPYAVALLSAMLWLYYALLTSDLLLLSINSIGCLVESLYLTVYLLYAPRQAMAFTLKLVCAMNLALFAAVVAALQLLVKATDRRVTLAGGIGASFALAVFVAPLTIIRQVIRTKSVEFMPFWLSFFLTLSAVVWFFYGLLMKDFFVATPNVLGLLFGLAQMVLYVVYKNPKKNSAVSEAAAAQQVEVKDQQQLQMQLQASPAVAPLDVDADADADLEAAAPATPQRPADDDAIDHRSVVVDIPPPPQPPPALPAVEVA

>OsSWEET13

MAGLSLQHPWAFAFGLLGNLISFTTYLAPIPTFYRIYKSKSTEGFQSVPYVVALFSAMLWIFYALIKSNEALLITINAAGCVIETIYIVMYLAYAPKKAKVFTTKILLLLNVGVFGVILLLTLLLSHGEQRVVSLGWVCVAFSVSVFVAPLSIIKRVIQSRSVEYMPFSLSLTLTLSAVVWFLYGLLIKDKYVALPNILGFTFGVVQMGLYVFYMNATPVAGEGKEGKGKLAAAEELPVVVNVGKLAAATPDRSTGAVHVHPVPRSCAAEAAAAEPEVLVDIPPPPPPRAVEVAAV

>OsSWEET14

MAGMSLQHPWAFAFGLLGNIISFMTYLAPLPTFYRIYKSKSTQGFQSVPYVVALFSAMLWIYYALLKSDECLLITINSAGCVIETIYIAVYLVYAPKKAKMFTAKLLLLVNVGVFGLILLLTLLLSAGDRRIVVLGWVCVGFSVSVFVAPLSIIRLVVRTKSVEFMPFSLSFSLTISAVVWFLYGLLIKDKYVALPNVLGFSFGVIQMGLYAMYRNSTPKAVLTKEVEAATATGDDDHSAAGVKEHVVNIAKLSAAVDVVKTREVHPVDVESPPAEAPPEEDDKAAAATAAAVAGAGEKKVAA

>OsSWEET15

MAFMSMERSTWAFTFGILGNLISLMVFLSPLPTFYRVYRKKSTEGFQSTPYVVTLFSCMLWMYYAFVKSGAELLVTINGVGCVIETVYLAMYLAYAPKSARMLTAKMLLGLNIGLFGVIALVTLLLSRGELRVHVLGWICVAVSLSVFAAPLSIIRLVIRTKSVEFMPFSLSFFLVLSAVIWFLYGLLKKDVFVALPNVLGFVFGVAQMALYMAYRSKKPLVASSSSAVVAAGLEIKLPEHVKEVQAVAKGAVAAAPEGRISCGAEVHPIDDVMPSEVVEVKVDDEETNRTDEMAGDGDHAMVRTEQIIKPDMAIVVEV

>OsSWEET16

MADPSFFVGIVGNVISILVFASPIATFRRIVRSKSTEEFRWLPYVTTLLSTSLWTFYGLHKPGGLLIVTVNGSGAALEAIYVTLYLAYAPRETKAKMVKVVLAVNVGALAAVVAVALVALHGGVRLFVVGVLCAALTIGMYAAPMAAMRTVVKTRSVEYMPFSLSFFLFLNGGVWSVYSLLVKDYFIGIPNAIGFALGTAQLALYMAYRRTKKPAGKGGDDDEDDEEAQGVARLMGHQVEMAQQRRDQQLRKGLSLSLPKPAAPLHGGLDRIIKSFSTTPIELHSILHQHHGGHHHHHRFDTVPDDDDEAVAAGGTTPATTAGPGDRH

*Zea mays*

>ZmSWEET1a

MEHIARFFFGVSGNVIALFLFLSPVVTFWRVIRKRSTEDFSGVPYNMTLLNCLLSAWYGLPFVSPNNILVSTINGTGSVIEAIYVVIFLIFAVDRRARLSMLGLLGIVASIFTTVVLVSLLALHGNARKVFCGLAATIFSICMYASPLSIMRLVIKTKSVEFMPFLLSLAVFLCGTSWFIYGLLGRDPFIIIPNGCGSFLGLMQLILYAIYRKNKGPAAPAGKGEAAAAAAEVEDTKKVAAAVELADATTNKAADAVGGDGKVASQV

>ZmSWEET1b

MEDVVKFVFGVSGNVIALFLFLSPVPTFWRIIRRKSTEDFSGVPYSMTLLNCLLSAWYGLPFVSPNNMLVSTINGAGAAIEAVYVVIFLAFASSQRTRLRMLGLASAVSAAFAAVALASMLALHGQGRKLMCGLAATVCSICMYASPLSIMRLVVKTKSVEYMPFLLSLAVFLCGTSWFVYGLLGRDPFVAIPNGCGSFLGAVQLVLYAIYRDSNSGGKQQAGDDVEMASDAKSSKKVADDVGGKEDRLV

>ZmSWEET2

MSSLYDVSCFAAGLAGNVFALALFLSPVPTFKRVLKAKSTEQFDGLPYLLSLLNCCICLWYGLPWVSDGGRALVATVNCTGALFQLAYISLFIFYADSRTTRLKVAGLLVLVVFAFALIAHASIAFFDQPLRQLFVGSVSMASLVSMFASPLAVMGVVVRTECVEFMPFYLSLSTFLMSASFAVYGLLLRDFFIYFPNGLGVILGAMQLVLYAYYSRRWKSSDSSAPLLA

>ZmSWEET3a

MVTSIRVIVGIIGSVVCVLLYAVPVLTFKRVVKEASVGEFSCVPYILALFSAFTWGWYGFPIVSDGWENLSLFGTCAVGVLFEASFVVVYVWFAPRDKKKSVVLMVSLVVATLCVIVSLSSFVFHTHHMRKQFVGSIGIVTSISMYSAPLVAVKQVILTKSVEFMPFYLSLFSLLTSFTWMLYGILGRDPYLTAPNGAGCLTGLLQIAVYCIYSRCNRPPKAVNGATTSREDANDCKV

>ZmSWEET3b

MLRLTFRWVIRKGNVEEFSCVPYILALLNCLLYTWYGLPVVSSGWENLPVATINGLGILLERFALQLVLPALALFGLTAALSSFAARTHRSRKAFVGSVGLVASVSMYTSPMVAAVSIYRSPNPLPPADFPLYFTASAGTKISGWSPTTPVDRYCMQKRVIATKSVEFMPFSLSLFSFLSSALWMAYGLLGRDLFIASPNFIGVPVGVLQLLLYCIYRRDHGAAAGAEAQAHGPAAAADQEKGMKAAAPVAVQPQEVAARVSEYK

>ZmSWEET4a

MISPDTIRTAIGVIGNGTALVLFLSPVPTFIRIWKKGSVEQYSPIPYVATLLNCMMWVLYGLPAVHPHSMLVITINGTGMAIQLTYVALFLLYSVGAARRKVVLLLAAEVGFVGAVAALVLSLAHTHERRSMVVGILCVLFGTGMYAAPLSVMKMVIQTKSVEYMPLFLSLASLVNGICWTAYALIRFDLYITIPNGLGVLFAVAQLVLYAIYYKSTQEIIEARKRKADQIAMTGVVVDGGKTNNQAGAGQY

>ZmSWEET4b

MMWVLYGLPLVHPHSMLVITINGTGMLIQLTYVALFLVYSAGAARRKVSLLLAAEVAFVGAVAALVLALAHTHERRSMVVGILCVLFGTGMYAAPLSVMKMVIQTKSVEYMPLFLSLASLVNGICWTAYALIRFDLYITIPNGLGVLFALAQLLLYAIYYKNTQKIVEARKRKAGQVAMTEVVVDGSRASNNNNNGGSGTY

>ZmSWEET4c

MVSADTIRTAIGVIGNGTALVLFLSPVPTFVGIWKKRAVEQYSPIPYVATLLNCMMWVLYGLPLVHPHSMLVVTINGTGMLIQLTYVALFILCSAGAVRRRVVLLFAAEVAFVVALAALVLTLAHTHERRSMLVGIVSVFFGTGMYAAPLSVMKLVIQTKSVEYMPLFLSLASLANSICWTAYALIRFDLYITIPNGLGVLFALGQLGLYAMFYKNTKQIMEARRRKADQQSTMMEVVTDASATPPPPPNNNNGGGGGNGY

>ZmSWEET6a

MISPDAARNVVGIIGNVISFGLFLSPVLTFWRIYKAKDVEEFKPDPYLATLLNCMLWVFYGIPVVHPNSILVVTINGIGLVIEAVYLTIFFLYSDSQKRKKAFAILAVEILFMVAVVLGVILGAHTHEKRSMIVGILCVIFGSMMYASPLTIMSRVIKTKSVEYMPFLLSLVSFLNGCCWTAYALIRFDLYVTIPNALGAFFGLVQLILYFCYYKSTPKKEKNVELPTVSSNVGGGNVTVSVER

>ZmSWEET6b

MISPDAARNVVGIIGNVISFGLFLSPVLTFWRICKARDVEEFKPDPYLATLLNCMLWVFYGIPVVHPNSILVVTINGVGLVIEAIYLTIFFLYSDGPKRRKAFGILAVEILFMVAVVLGVILGAHTHEKRSMIVGILCVIFGSMMYASPLTIMSRVIKTKSVEYMPFLLSLVSFLNGCCWTAYALIRFDLYVTIPNALGAFFGLIQLILYFCYYKSTPKEKNVELPTVSSNAGGGNVTVSVER

>ZmSWEET10

MAGGLFSMEHPWASVFGILGNIISFLVFLAPVPTFLRVYRKKSTEGFSSVPYVVALFSCTLWILYALVKTNSSPLLTINAFGCVVEAAYILLYLVYAPRGARLRALASFLLLDVAAFSLVAVVTVVLVAEPHRVRVLGSVCLAFSMAVFVAPLSVIFVVIRTKSAEFMPFTLSFFLTLSAVAWFLYGLFTKDPYVTLPNVGGFFFGCIQMVLYCCYRKRKPASVVVLPTTTAAAAVAQQLEAEMELPLAAHQHQLAVAVLPTCAAPVLAELQKLEEAMGS

PRKGGVKAI

>ZmSWEET11

MAGGFFSMAHPAVTLSGIAGNIISFLVFLAPVATFLQVYRKKSTGGFSSVPYVVALFSSVLWIFYALVKTNSRPLLTINAFGCGVEAAYIVLYLAYAPRRARLRTLAYFFLLDVAAFALVVAVTLFAVREPHRVKFLGSVCLAFSMAVFVAPLSIIVKVVKTKSVEFLPISLSFCLTLSAVAWFCYGLFTKDPFVMYPNVGGFFFSCVQMGLYFWYRKPRPAAKNNAVLPTTTDGANAVQVQGQVIELAPNTVAILSVSPIPIVGVHKIEVVEQQHKEAAVAAETRRMAAANPDGAMPEVIEIVPAAAAV

>ZmSWEET12a

MITVGHPVVFAVGILGNILSFLVTLAPVPTFYRVYKKKSTESFQSVPYVVALLSAMLWLYYALLSVDLLLLSINTIACVVESVYLAIYLTYAPKPAMAFTLKLLCTMNMGLFGAMVAFLQFYVDGQRRVSIAGGVGSAFAFAVFRQVIRTKSVEFMPFWLSFFLTVSAVAWFFYGLLMKDFFVAMPNVLGLLFGLAQMALYFVYRNRNPKKNGAVSEMQQAAAVQADAEKEQQLRQADADADADGKAATTDDDGGQTAVVVDIMPPPPLLPAERAPPLPLPPHPAMVMTTAHQTAVEV

>ZmSWEET12b

MITVGHPVAFAVGILGNILSFLVILAPVPTFYRVYAKKSTESFQSVPYVVALLSATLWLYYALLSTDLLLLSINTVACVAESVYLAVYLAYAPGPAKAFTLKLLCAINMGLFGAMVAFLQFYVVDTQRRVSIAGGVGAAFALAVFVAPLAIIRRVMRTKSVEFMPFWLSFFLTVSAVVWFFYGLLIKDFFVAMPNVLGLLFGLAQMVLFFVYRNRNPKKNGAVSEMQQAAVQADAEKERRSHANADGEADVRTVIVDIMPPPPAMMVMMTAAHQTPPAVEVV

>ZmSWEET13a

MAGMSLQHPWAFAFGLLGNVISFMTFLAPIPTFYRIYKSKSTEGFQSVPYVVALFSAMLWIFYALIKSNETFLITINAAGCVIETIYVVMYFVYAPKKAKLFTAKIMVLLNGGVFGVILLLTLLLFKGSKRVVLLGWICVGFSVSVFVAPLSIMRRVIQTKSVEYMPFSLSLSLTLSAVVWFLYGLLIKDKYVALPNILGFTFGVVQMVLYVLYMNKTPVAATAEGKDAGKLSSAADEHVLVNIAKLSPALPERSSGVHPVTQMAGVPVRSCAAEATAPAMLPNRDVVDVFVSRHSPAVHVA

>ZmSWEET13b

MAGLSLEHPWAFAFGLLGNVISFMTFLAPIPTFYRIYKSKSTEGFQSVPYVVALFSAMLWIFYALIKSNETFLITINAAGCVIETIYIVMYFVYAPKKAKLFTAKIMALLNGGVFGVILLLTLLLFKGSKRVVLLGWICVGFSVSVFVAPLSIMRRVIQTKSVEYMPFSLSLSLTLSAVVWFLYGLLIKDKYVALPNVLGFIFGVVQMVLYVFYMNKTPVAAAVGKDAGKLPSAADEHVLVNIAKLNPALPERTSGMHPVTQMAAVPARSCAAEAIAPAMLPNRDVVDVFVSRHSPAVHVV

>ZmSWEET13c

MAGLSLQHPWAFTFGLLGNVISFMTFLAPIPTFYRIYKSKSTEGFQSVPYVVALFSAMLWIFYALIKSNETFLITINAAGCVIETVYVVMYFVYATKKGRMFTAKIMLLLNVGAFGAILLLTLLLFKGDKRVVMLGWICVGFSVSVFVAPLSIMRRVIQTKSVEYMPFSLSLSLTLSAVVWFLYGLLIKDKYVALPNILGFTFGVVQMVLYVVYMNKTPLPVADGKAAGKLPSAADEHVVVNVTKLSPGRLPPVTQMAAVPTKSCATEAAAPATLPSRDVVDVLVNRHSPAVHVT

>ZmSWEET14a

MAGLSLQHPMAFAFGLLGNIISFMTYLAPLPTFCRIYRNKSTEGFQSVPYVVALFSAMLWIYYALLKSNEFLLITINSAGCVIETLYIATYLLYAPNKAKLFTAKILLLLNVGVFGLILLLTLLLSAGPHRVVVLGWVCVAFSVSVFVAPLSIIRQVVRTRSVEFMPFSLSFSLTASAVVWFLYGLLIKDKYVALPNVLGFTFGVVQMGMYALYRNATPRVPAAKEAAAAADDGNTFNFKAPGEHVVTIAKLTAAAPATAAELIIKARDDAQHPPEEEAAAAKAAPAKSKLLIPLPEHAYACMCIIRSGSHHKLGRACLLGTSTRPPACLPARMIQSSCYIRKG

>ZmSWEET14b

MAGLSLLHPMAFAFGLLGNIISFMTYLAPLPTFYRIYKNKSTEGFQSVPYVVALFSAMLWIYYALLKSNELLLITINSAGCVIETLYIAMYLLYAPKKAKLFTAKILLLLNVGVFGLILLLTLLLSAGQRRVVVLGWVCVAFSVSVFVAPLSIIRQVVRTRSVEFMPFSLSLSLTVSAVVWFLYGLLIKDKYVALPNVIGFSFGVVQMGLYALYRNATPRVPAKDVADDASKDKAPGEHVVVTIAKLTAATTAPAAAVAEDLVKVHDGHPEEAAKGAAKPAENGAGRSDAEQV

>ZmSWEET15a

MAFLNMEQQTWAFTFGILGNIVSLMVFLSPLPTFYRVYRNKSTEGFQSTPYVVTLFSCMLWILYALLKPGAELLVTINGVGCVVETVYLAMYLVYAPKAARVLAAKMLLGLNVAVFGLVALVTMLLSDAGLRVHVLGWICVSVSLSVFAAPLSIMRQVIRTKSVEFMPISLSFFLVLSAVVWFAYGALKKDVFVAFPNVLGFVFGLAQMALYMAYRKPAAALVIIPEQSKEEVAEGKASCGGAEVHPIDIAEVHDLQTVVVDVDVEPVTYAAASGMVDGSVGRPRAPEELVIKPDMVTVIAAEA

>ZmSWEET15b

MAFLNMEQQTWAFTFGILGNIISLMVFLSPLPTFYRVYRKKSTEGFQSTPYVVTLFSCMLWIFYALLKSGAELLVTINGVGCVIEAAYLAAYLVYAPKAARALTAKMLLGLNVGVFGLAALATMVVSSAGLRVRVLGWICVSVALSRQVVRTKSVEFMPISLSFFLVLSAVIWFAYGALKRDVFVAFPNVLGFVFGVAQIALYMAYRNKEPAAVTVEEAKLPEHAKEVVVAAAAAEARASCGAEVHPIDIDIEATPTPVEEVHEPQVVVVVDVDVEPVTCAGAAEAAAGAGADASGVADGGVPGPMAPPEQLAIKPDMAISVEA

>ZmSWEET16

MADPSFLVGIVGNVISILVFASPIATFRRIVRSRSTEDFRWLPYVTTLLSTSLWTFYGLLKPGGLLVVTVNGAGAALEAAYVALYLVYAPRETKAKMAKVVVAVNVAFLAAVVAVALLALHGGARLFAVGLLCAALTVGMYAAPLGAMRTVVKTRSVEYMPFSLSFFLFLNGGVWSIYSLLVKDYFIGVPNAIGLVLGTAQLLLYLAYRKAPASKDDDEEAAAAASGDDGDDEEEEEGLTHLMGQQVEMAQRGRLRLHKGQSLPKPPPGGPLSSPRHGFGSIIKSLSATPVELHSVLYQHARFQPVKKDDDDVEAND

>ZmSWEET17a

MDSTLFIIGVIGNIISVLVFISPIKTFWRIVRSGSTEEFEPAPYVFTLLNALLWLYYGATKPDGLLVATVNGFGAAMEAIYVVLFIVYAANHATRVKTAKLAAALDIGGFGVVFVATTFAINELNMRIMVIGMICACLNKTVITTKSVEFMPFFLSFFLFLNGGIWATYAVLDRDIFLGIPNGIGFILGTIQLIIYAIYMNSKVSQSSKEIASPLLASSQEEAASHV

>ZmSWEET17b

MDSTLFIIGVIGNIISVLVFISPIKTFWRIVRGGTTEEFEPAPYVLTLLNALLWLYYGLTKPDGFLVATVNGFGAVMEAIYVVLFIVYAANHATRVKTAKLAAALDIGGFGVVFAATTFAISEFELRIMVIGMICACLNVLMYGSPLASMKTVITTKSVEFMPFFLSFFLFLNGGVWATYAVLDRDIFLGIPNGIGFVLGTIQLIVYAIYMNSKASQCSKETASSPLLASDRGEASSHV

*Arabidopsis thaliana*

>AtSWEET1

MNIAHTIFGVFGNATALFLFLAPSITFKRIIKNKSTEQFSGIPYPMTLLNCLLSAWYGLPFVSKDNTLVSTINGTGAVIETVYVLIFLFYAPKKEKIKIFGIFSCVLAVFATVALVSLFALQGNGRKLFCGLAATVFSIIMYASPLSIMRLVVKTKSVEFMPFFLSLFVFLCGTSWFVYGLIGRDPFVAIPNGFGCALGTLQLILYFIYCGNKGEKSADAQKDEKSVEMKDDEKKQNVVNGKQDLQV

>AtSWEET2

MDVFAFNASLSMCKDVAGIAGNIFAFGLFVSPMPTFRRIMRNKSTEQFSGLPYIYALLNCLICLWYGTPFISHSNAMLMTVNSVGATFQLCYIILFIMHTDKKNKMKMLGLLFVVFAVVGVIVAGSLQIPDQLTRWYFVGFLSCGSLVSMFASPLFVINLVIRTKSVEFMPFYLSLSTFLMSASFLLYGLFNSDAFVYTPNGIGTILGIVQLALYCYYHRNSIEEETKEPLIVSYV

>AtSWEET3

MGDKLRLSIGILGNGASLLLYTAPIVTFSRVFKKKSTEEFSCFPYVMTLFNCLIYTWYGLPIVSHLWENLPLVTINGVGILLESIFIFIYFYYASPKEKIKVGVTFVPVIVGFGLTTAISALVFDDHRHRKSFVGSVGLVASISMYGSPLVVMKKVIETRSVEYMPFYLSFFSFLASSLWLAYGLLSHDLFLASPNMVATPLGILQLILYFKYKNKKDLAPTTMVITKRNDHDDKNKATLEFVVDVDRNSDTNEKNSNNASSI

>AtSWEET4

MVNATVARNIAGICGNVISLFLFLSPIPTFITIYKKKKVEEYKADPYLATVLNCALWVFYGLPMVQPDSLLVITINGTGLAIELVYLAIFFFFSPTSRKVKVGLWLIGEMVFVGIVATCTLLLFHTHNQRSSFVGIFCVIFVSLMYIAPLTIMSKVIKTKSVKYMPFSLSLANFLNGVVWVIYALIKFDLFILIGNGLGTVSGAVQLILYACYYKTTPKDDEDEEDEENLSKVNSQLQLSGNSGQAKRVSA

>AtSWEET5

MTDPHTARTIVGIVGNVISFGLFCAPIPTMVKIWKMKSVSEFKPDPYVATVLNCMMWTFYGLPFVQPDSLLVITINGTGLFMELVYVTIFFVFATSPVRRKITIAMVIEVIFMAVVIFCTMYFLHTTKQRSMLIGILCIVFNVIMYAAPLTVMKLVIKTKSVKYMPFFLSLANFMNGVVWVIYACLKFDPYILIPNGLGSLSGIIQLIIYITYYKTTNWNDDDEDKEKRYSNAGIELGQA

>AtSWEET6

MVHEQLNLIRKIVGILGNFISLCLFLSPTPTFIHIVKKKSVEKYSPLPYLATLLNCLVRALYGLPMVHPDSTLLVTISGIGITIEIVFLTIFFVFCGRQQHRLVISAVLTVQVVFVATLAVLVLTLEHTTDQRTISVGIVSCVFNAMMYASPLSVMKMVIKTKSLEFMPFLLSVVGFLNAGVWTIYGFVPFDPFLAIPNGIGCVFGLVQLILYGTYYKSTKGIMEERKNRLGYVGEVGLSNAIAQTEPENIPYLNKRVSGV

>AtSWEET7

MVFAHLNLLRKIVGIIGNFIALCLFLSPTPTFVRIVKKKSVEEYSPIPYLATLINCLVWVLYGLPTVHPDSTLVITINGTGILIEIVFLTIFFVYCGRQKQRLIISAVIAAETAFIAILAVLVLTLQHTTEKRTMSVGIVCCVFNVMMYASPLSVMKMVIKTKSVEFMPFWLSVAGFLNAGVWTIYALMPFDPFMAIPNGIGCLFGLAQLILYGAYYKSTKRIMAERENQPGYVGLSSAIARTGSEKTANTNQEPNNV

>AtSWEET8

MVDAKQVRFIIGVIGNVISFGLFAAPAKTFWRIFKKKSVEEFSYVPYVATVMNCMLWVFYGLPVVHKDSILVSTINGVGLVIELFYVGVYLMYCGHKKNHRRNILGFLALEVILVVAIILITLFALKGDFVKQTFVGVICDVFNIAMYGAPSLAIIKVVKTKSVEYMPFLLSLVCFVNAGIWTTYSLIFKIDYYVLASNGIGTFLALSQLIVYFMYYKSTPKEKTVKPSEVEISATERV

>AtSWEET9

MFLKVHEIAFLFGLLGNIVSFGVFLSPVPTFYGIYKKKSSKGFQSIPYICALASATLLLYYGIMKTHAYLIISINTFGCFIEISYLFLYILYAPREAKISTLKLIVICNIGGLGLLILLVNLLVPKQHRVSTVGWVCAAYSLAVFASPLSVMRKVIKTKSVEYMPFLLSLSLTLNAVMWFFYGLLIKDKFIAMPNILGFLFGVAQMILYMMYQGSTKTDLPTENQLANKTDVNEVPIVAVELPDVGSDNVEGSVRPMK

>AtSWEET10

MAISQAVLATVFGILGNIISFFVCLAPIPTFVRIYKRKSSEGYQSIPYVISLFSAMLWMYYAMIKKDAMMLITINSFAFVVQIVYISLFFFYAPKKEKTLTVKFVLFVDVLGFGAIFVLTYFIIHANKRVQVLGYICMVFALSVFVAPLGIIRKVIKTKSAEFMPFGLSFFLTLSAVMWFFYGLLLKDMNIALPNVLGFIFGVLQMILFLIYKKPGTKVLEPPGIKLQDISEHVVDVVRLSTMVCNSQMRTLVPQDSADMEATIDIDEKIKGDIEKNKDEKEVFLISKN

>AtSWEET11

MSLFNTENTWAFVFGLLGNLISFAVFLSPVPTFYRIWKKKTTEGFQSIPYVVALFSATLWLYYATQKKDVFLLVTINAFGCFIETIYISMFLAYAPKPARMLTVKMLLLMNFGGFCAILLLCQFLVKGATRAKIIGGICVGFSVCVFAAPLSIIRTVIKTRSVEYMPFSLSLTLTISAVIWLLYGLALKDIYVAFPNVLGFALGALQMILYVVYKYCKTSPHLGEKEVEAAKLPEVSLDMLKLGTVSSPEPISVVRQANKCTCGNDRRAEIEDGQTPKHGKQSSSAAAT

>AtSWEET12

MALFDTHNTWAFVFGLLGNLISFAVFLSPVPTFYRICKKKTTEGFQSIPYVVALFSAMLWLYYATQKKDVFLLVTINSFGCFIETIYISIFVAFASKKARMLTVKLLLLMNFGGFCLILLLCQFLAKGTTRAKIIGGICVGFSVCVFAAPLSIIRTVIKTKSVEYMPFSLSLTLTISAVIWLLYGLALKDIYVAFPNVIGFVLGALQMILYVVYKYCKTPSDLVEKELEAAKLPEVSIDMVKLGTLTSPEPVAITVVRSVNTCNCNDRNAEIENGQGVRNSAATT

>AtSWEET13

MALTNNLWAFVFGILGNIISFVVFLAPVPTFVRICKKKSTEGFQSLPYVSALFSAMLWIYYAMQKDGTAFLLITINAFGCVIETIYIVLFVSYANKKTRISTLKVLGLLNFLGFAAIVLVCELLTKGSTREKVLGGICVGFSVSVFAAPLSIMRVVVRTRSVEFMPFSLSLFLTISAVTWLFYGLAIKDFYVALPNVLGAFLGAVQMILYIIFKYYKTPVAQKTDKSKDVSDHSIDIAKLTTVIPGAVLDSAVHQPPALHNVPETKIQLTEVKSQNMTDPKDQINKDVQKQSQV

>AtSWEET14

MVLTHNVLAVTFGVLGNIISFIVFLAPVPTFVRICKKKSIEGFESLPYVSALFSAMLWIYYALQKDGAGFLLITINAVGCFIETIYIILFITYANKKARISTLKVLGLLNFLGFAAIILVCELLTKGSNREKVLGGICVGFSVCVFAAPLSIMRVVIRTKSVEFMPFSLSLFLTISAITWLFYGLAIKDFYVALPNILGAFLGAVQMILYVIFKYYKTPLVVDETEKPKTVSDHSINMVKLSSTPASGDLTVQPQTNPDVSHPIKTHGGDLEDQMDKKMPN

>AtSWEET15

MGVMINHHFLAFIFGILGNVISFLVFLAPVPTFYRIYKRKSTESFQSLPYQVSLFSCMLWLYYALIKKDAFLLITINSFGCVVETLYIAMFFAYATREKRISAMKLFIAMNVAFFSLILMVTHFVVKTPPLQVSVLGWICVAISVSVFAAPLMIVARVIKTKSVEYMPFTLSFFLTISAVMWFAYGLFLNDICIAIPNVVGFVLGLLQMVLYLVYRNSNEKPEKINSSEQQLKSIVVMSPLGVSEVHPVVTESVDPLSEAVHHEDLSKVTKVEEPSIENGKCYVEATRPETV

>AtSWEET16

MADLSFYVGVIGNVISVLVFLSPVETFWRIVQRRSTEEYECFPYICTLMSSSLWTYYGIVTPGEYLVSTVNGFGALAESIYVLIFLFFVPKSRFLKTVVVVLALNVCFPVIAIAGTRTLFGDANSRSSSMGFICATLNIIMYGSPLSAIKTVVTTRSVQFMPFWLSFFLFLNGAIWGVYALLLHDMFLLVPNGMGFFLGIMQLLIYAYYRNAEPIVEDEEGLIPNQPLLA

>AtSWEET17

MAEASFYIGVIGNVISVLVFLSPVETFWKIVKRRSTEEYKSLPYICTLLGSSLWTYYGIVTPGEYLVSTVNGFGALVETIYVSLFLFYAPRHLKLKTVDVEAMLNVFFPIAAIVATRSAFEDEKMRSQSIGFISAGLNIIMYGSPLSAMKTVVTTKSVKYMPFWLSFFLFLNGAIWAVYALLQHDVFLLVPNGVGFVFGTMQLILYGIYRNAKPVGLSNGLSEIAQDEEEGLTSRVEPLLS

*Vitis vinifera*

>VvSWEET1

MDAHHALHFTFGIFGNATALFLFLAPLITFKRIIKSKSTEQFSGIPYVMTLLNCLLSAWYGLPFVSKNNILVSTINGTGAAIEIIYVLIFIAYSIKKERAKILGLFIFVLSVFGVVVFVSLFALHGHSRKLFCGLAATIFSIIMYASPLSIMRMVIKTKSVEYMPFFLSLFVFLCGTSWFVFGLLGKDPFVAVPNGFGCGLGAMQLILYAIYCKKGKSKNLAAADKPVDMELGKPQQEKQSRAQNGNVQ

>VvSWEET2a

MSRSLLLPVNTICKDAAGVAGNIFAFGLFVSPIPTFRRIARNRSTESFSGLPYIYALLNCLVTLWYGTPLVSYNNIMVTTVNSMGAAFQLVYIILFITYTDKRKKVRMFGLLMVDIVLFLVIVVGSLEISDFTIRRMVVGFLSCAALISMFASPLFVINLVIQTRSVEFMPFYLSLSTFLMSASFLAYGILNNDPFVYVPNGAGTVLGIVQLGLYSYYKRTSAEESREPLIVSYGQ

>VvSWEET2b

MSSVYSVCCDAAGIAGNLSAFVLFVSPIPTFRRIIRNGSTEQFSGLPYIYALLNCLICLWYGMPLVSPGIILVATVNSVGAIFQLIYIGIFITFAEKAKKMKMSGLLTAIFGIYAIIVFASMKLFDPHARQLFVGYLSVASLISMFASPLFIINLVIRTRSVEYMPFYLSLSTFLMSLSFFTYGMFKHDPFIYVPNGIGTILGVVQLVLYAYYSRTSTEDLGLRESFIESYA

>VvSWEET3

MGDRLHLAIGVMGNAASLLLYTAPILTFARVMRKKSTEEFSCIPYIIALLNCLLYTWYGLPVVSYRWENFPVVTINGLGILLEFSFILIYFWFTSPRGKIKVVGTVVPVVTVFCITAIISSFVLHDHHHRKMFVGSVGLVASVAMYGSPLVVVRQVILTKSVEFMPFYLSFFSFLTSFLWMAYGLLGHDLLLASPNLVGSPLGILQLVLYCKYRKRGIMEEPNKWDLEGNDEKSKQLQPVINNDSNGKI

>VvSWEET4

MTGADTARTVIGIIGNVISFALFASPSPTFWRIWKKRSVEEFSPDPYLATVMNCMFWIFYGLPVVHPNSTLVVTINSIGLAVELIYLTIYFVFAPNKGRLKVIGVLCLELAFMAAVVVVTLTKLHTHASRSNLVGIFCVVFGVLMYASPLTVMKKVITTKSVEYMPFYLSLTNFLNGVIWLTYALIQFDLYITIGNGLGAVSGAIQLILYACYYKSTPKDKEGKEKGKSSEVELASPKRLNKPTPQATAANTAA

>VvSWEET5a

MVNPDTIRTIVGIIGNVISFGLFASPIPTFIQIVKKKTVGEFKPDPYLATVLNCMMWVLYGLPFVRPDSLLVITINGGGLVIELIYVTIFFVYADSLKRKKIALWLLFEVIFMAIIAAITMLLFHGTKNRSLFVGLLCVVFNVIMYASPLTVMRQVIRTKSVKYMPFTLSLANFANGIVWSIYALIKFDPYILIPNGLGSLSGAVQLILYATYYKSTPKDEEDKKPPEVQLSGM

>VvSWEET5b

MVSKDTARTIVGIIGNIISFGLFASPIPTFKKIYHEKTVGGFKPDPYLATVLNCSLWVLYGLPFVHPDSVLVITINGIGLVMEIIYVSIFFTYSDWAKRKKIVMALLCIVIFVAAVAGITMGAFHTHHDRSMFVGILCVVFNVVMYASPLTVMRRVIRTRSVKYMPFFLSLANLMNGIVWLIYALIKIDAYIVIPNALGTISGLVQMVLYAAFYKSTPREEEEVKKTQEVQLSGIQ

>VvSWEET7

MSSTEVARTAVGILGNIIALFLFLSPVPTFISIWKKGSVEQYSPVPYLATFINCMVWVLYGLPMVHPHSTLVVTINGTGFVIELVYLILFIVFSNRGNRLRVIMIALVEIIFVAIVALLTLTMVHTTDRRSMIVGTICILFNIMMYASPLSVMKMVIRTKSVEYMPFFLSLAAFGNGIAWTTYALIRFDLFITVPNGLGTLFAAAQLTLYAMFYKSTKRQLAERKQGKVEMDLAQVVVTAEPMDKAQNGGGGGVHEVRRT

>VvSWEET9

MAVVTVKQLAFIFGLLGNLVSFMVYLSPVPTFFKIYKRKTSEGYQALPYSVGLLCASLFLYYALLQSGKFLILSINTIGSTIQATYLVLFIIYSPRAGKVATLKMILILNVASLGLVLLLTTLFSKGKTRIQVVGWISAGVNIGTFVAPLSIIKRVIETRSVEYMPFNLSFFLTICATMWFFYGIFVRDFFIAIPNVVGFVFGIAQMFLYIIYKYMMKSDETTLEQLEETTERPLYVPTANHEPSGQELKAVTITSPRQVDYFTEHHPMFMERDEYLSQ

>VvSWEET10

MALFPIHHPLVFIFGILGNLISFMVYLAPLPTFYQIYKRKSTEGFQSVPYVVALFSAMLWIYYAFLNTDASLLITINSVGCVIETSYIVMFLVYAPKKARITTVKLVFLMNICGFGSILLLTLLLAEGANRVRILGWVCLVFSLSVFLAPLCIMRQVIRTKSVEYMPFLLSFFLTLSAVMWFFYGLMLKDFYIAGPNILGFVFGIVQMVLYLIYRNRKKVLENEKLPELSEQIIDVVKLSTMVCSEVNLTNQQHSNEGHGTTGLEVIVALQ

>VvSWEET11

MAMLTVPHMAFAFGILGNIVSFLVYLSPLPTFYRIYKRKSTEGFQSIPYSVALFSAMLLLYYAFLKTDNQIMLITINSVGTCIEATYLLVYMIYAPRTAKIYTAKLLLLFNTGVYGAIVLSTFFLSKGHRRAKIVGWVCAAFSLCVFAAPLSIMRLVIRTKSVEYMPFPLSFFLTICAVMWFFYGLLIRDFYIAFPNILGFAFGIAQMILYTIYKNAKKGVLAEFKLQELPNGLVFPTLKKAENTDTNPNDQPEDTAMTEGGARDKAVEPSGELKHNSSSLVVRFCLRALRLSFHHVSFSRIIAYTNQRNTVNTMRVYLLYIAMYENKSILVFITLFSQILQ

>VvSWEET12

MAMFTVGHHPWVFASGILGNLMSFLVYLAPIPTFTRVIKKKSTEGFQSVPYVIALFSAMLWMYYGLVNTNASFLLSVNGFGCFIEIIYISIYLIFAPRRARILTLRLLLLINLGAFCLILIVTNFMVKRPHRVKAVGWVCLIFAVSVFAAPLSIMASILYRLVIRTKSVEFMPLPLSICLTLSAVGWFFYGILQMDLYIAMPNTLGFVFGLIQMILYAMYRNSTPVTKEPKLPEQVIDIVKLNTNSTPEVHPVSTLQPNCVENEGGNGQNARKETEHAEESMGGSNRV

>VvSWEET15

MAMAMANHHTLGLIFGILGNIISFLVYFAPAPTFYRIYKRKSAEGFHSLPYIVALFSAMLWLYYALLKKDAFLLITINSFGCAIESFYILLYFFYAPMQAKKQTLKVVISLNVGVFSILVVLIQFLLKGSNRINVFGWICASFSVAVFAAPLSIVAKVIRTKSVEFMPFSLSFFLTLSAIMWFAYGLLKNDPCVAIPNILGVILGLVQMVLYGFYRNAGKEKMEKKLPEHIIDMVMLSTLGTSDIHPIGAQQNGIKKSGSEDVKDDEETGNREKSTENSGELQPNGSTV

>VvSWEET16

MASLSFIIGIIGNVISILVFASPIGTFRRVVKKKSTENYKGIPYITTLLSTSLWSFYGILKPGGLLVLTVNGAGAIMQFIYVTLFLIYAPRDVKIKSMKVAAVLDVGFLGAVIALTLLAFHGSSRLICVGIFCAGLTIVMYASPLSAMRMVIKTKSVEFMPFFLSFFLFLNGGVWSVYAVLVTDFFIGVPNAVGFVLGSAQLILYAVYRNKSRPSATSEERVEEEGSAHTVKRAVEMQVSKDDGKASPKNHSLSKGRSLPMPFISRQYSLQKIMRTLSWSPCELQDRQQDKDIEKGDI

>VvSWEET17a

MESLSFFAGVIGNIISVLVFLAPIGTFWRIVKHRSTQDFESLPYVCTLLNSSLWTYYGIIKPGEILVATVNGFGVVVEAAYVTLFLIYAPAKMRAKTVALVSLLDVGFLAAAILVTRLALQGDTRIDALGFICSGLNIVMYGSPLAAMKTVVTTKSVEFMPFFLSFFLFLNGGIWTIYAVLVRDYFLAVPNGTGLVLGTAQLVLYAIYRNSKPSNKFSIEDGSQEEHLIASSS
