# Supplementary material for: Genome-wide analysis of the SWEET gene family in Hemerocallis citrina and functional characterization of HcSWEET4a in response to salt stress
Source: BMC Plant Biol. 2024 Jul 11;24:661. doi: 10.1186/s12870-024-05376-y (PMC11238388; doi:10.1186/s12870-024-05376-y)
Supplement: Supplementary file 2 — Supplementary Material 2 [file 12870_2024_5376_MOESM2_ESM.docx]

**Supplementary Information 2** CDS sequences of 19 *HcSWEET* genes

> HcSWEET1a

ATGGAAACTGTTCTGCATTTTGTGTTTGGGATGTTTGGGAATATTACTGCTCTTTTCCTCTTCTTTTCACCTATCGTGACGTTTCGGAGGATCATAAAGAATCGATCTACTGAGGAGTTCTCCGGGGTCCCCTACAACATGACGATGCTCAACTGCCTGTTATCTGCTTGGTATGGGCTGCCTTTTGTTTCGCCAAACAACCTCCTGGTGTGGACGATAAATGGCACCGGAGTGCTTATTGAGGCAATCTATGTGCTAATATTCCTGATATTTGCACCGAAGAAGGTGAGGAGTCGAATGATGGGACTCCTGGCGCTTGTGTCTTCTGTGTTCATCTCGGTTGCACTCATCTCGGTGCTGGCTCTTCACGGACAGAGTCGTAAGGTCTTCTGTGGGCTTGCAGCCACGATCTTCTCCATCTGCATGTACGGCTCGCCTTTGTCTATTATGAGGTTGGTGATCCGAACAAAAAGCGTGGAGTACATGCCATTCTTTCTCTCCCTCTTCGTGTTTCTGTGCGGGACCTCTTGGTTCATCTACGGCCTTCTTGGTCATGATGTTTTCATTGCGGTACCAAACGGGTGTGGTAGCGCGCTTGGATTAGTGCAGCTAATCCTTTATGCAATTTACAGGAATCATAAGGGGAATAAAAGTAGTGACACTAACGGAGAGTCCCTTCAGATGACCGATGTGAAGCCTACTGACAACTATGCGAAAAATGTCGAACAAGTATAG

> HcSWEET1b

ATGGAGAATGTTCTGCATTTTGTGTTTGGAATCTTTGGGAATGTTACTGCTCTGTTCCTCTTCTTTTCACCTATGGTGACGTTTCGGAGGATCATAAAGAATCGATCTACTGAGGAGTTCTCCGGGGTCCCCTACAACATGACCATGCTCAACTGCCTGTTATCTGCTTGGTATGGGCTGCCTTTTGTGTCACCAAACAACCTCCTGGTTTGGACGATAAATGGCACCGGAGCGCTTATTGAGGCTATCTACGTGCTGATATTCCTGATATTTGCGCCGAAGAAGGTGAGGGGTCGAATGATGGGACTCCTGGCGCTTGTGTCTTCTGTGTTCATCTCGGTTGCACTCATCTCAGTGCTGGCTCTTCACGGACAGACTCGGAAGTTCTTCTGTGGCTTTGCAGCCACGATCTTCTCCATCTGCATGTACGGCTCGCCTTTGTCTATTATGAGGCTGGTGATCCGAACAAAAAGTGTGGAGTACATGCCCTTCTTTCTCTCTCTCTTCGTGTTTCTGTGCGGGACCTCTTGGTTCATCTATGGCCTTCTTGGTCGCGATGTTTTCATTGCGGTACCGAACGGGTGTGGTAGCGCACTTGGATTAGTGCAGCTAATCATTTACGCAATTTACAGGAATCATAAAGGGGATAAGAGTACTGACACTAACGGAGAGTCTCTTCAGATGACCGATGTGAAGCCTACTGACAACTATGAGAAAAATGTGAAGCCTACTAACAACTATGAGAAAAATGTTGAAGAAGTTTAG

> HcSWEET2

ATGGGGCCTTCTTCTTTCTATGAACTCTGCACCTATGCAGCTGGAATTGCTGGCAATCTCTTTGCCTTTGTGCTCTTTGTCTCACCACTACCCACATTCAGAAGAATTATAAGAAACCGATCAACTGAGCAATTCTCAGGGTTGCCTTATGTGTACTCCCTCTTGAACTGTTTGATTTGCTTCTGGTATGGACTACCTTTTGTGTCCCGAGGTGTGATCTTGATGTGCACAGTCAATTCAGTTGGTGCCGCGTTCCAACTAGTTTATATCACCGTGTACATTCTATTCGCTGATTCCTCAGGAAAGGTGAAGATATCTGGACTTCTCATTGCTGTTTTCGGTGCTTTTGCCCTCATTATGTATATTACCCTCCAGTATTTTGACCATGGAATGAGGAAAATGGTTGTTGGGTATTTGAGTATAGCTACTCTTATTTCAATGTTTGCATCACCGTTATCTGTAATCAACTTGGTGATTCGGACAAAGAGTGTCGAGTTCATGCCCTTCTACCTATCACTTGCAACCTTCTTGATGAGCATCTCATTTTTTGCTTACGGGATGCTACTGCAAGACTTCTTCGTATATCTCCCAAATGGGATAGGATCTATCTTGGGTGCCATACAATTACTTTTATATGCTTACTACAGCAAGAATGTGAGCAGAGATACTAGATTACCATTGCTGCAATGA

> HcSWEET3

ATGGGGACAAGCTTGCGCTTTCCAGTTGGAATATTGGGGAATGCGGCTTCCTTGTTCCTTTACACAGCACCCATATTAACCTTTGCAAGGGTGATAAGGAAGAGGAGTACAGAGGAGTTCTCATGCATACCTTACATCATTGCTCTTCTGAATTGCCTTCTATATACTTGGTTTGGTTTGCCAGTGGTAAGCAAAGGCTGGGAGAACTTCACAGTGGCTACAATCAATGGCTTAGGGATATTACTGGAGATCTCATTCATTTTAATCTATATATGGTTTGCTTTAGCAAAACGTAAGAAATTCGCAATATCAATGTTGGTGGCGGTAATAGTTGTATTTGGGATGACTGCATTTGTATCCAGTGTAGTGTTGCATGACCACCCGCATCGAAAAGTATTTGTTGGAAGTGTGGGACTTGTAGCCTCTGTAGCAATGTATAGCTCTCCACTGGTAGCTATGAGACTAGTGATAAAGACGAAAAGCGTGGAGTTCATGCCATTCTACCTATCCTTCTTCTCTTTTCTAGCCAGTTCACTCTGGATGTTATATGGGCTATTAGGCCAAGAGCTTTTCATTGCGGCACCTAATTTCTTGGGCACCCCTGTGGGAATCCTTCAGCTTATACTATACTGCATGTACAAGAAAAAGAAAGGAGGTCACCAAGAACCTAAGAATATAGACGTAGAGAATAATGGAGAAAAGCAGCTGAACTCATGA

> HcSWEET4a

ATGGTTTCAGCCGACACCATCCGAACTTGTGTTGGAATCCTAGGAAATTTGATAGCTCTAGCCCTATTTTTATCACCAGTGCCAACTTTTTGCAGGATTTGGAAGAAAGGTTCAGTTGAGCAATTCTCAGCAGTGCCATACCTGGCAACTCTGCTCAACTGCATGCTTTGGGTGGTGTACGGCCTCCCTTTGGTTCACCCACACAGCACTTTGGTCTTGACCATCAATGGCTCTGGGTTGATTATTGAGCTCTCCTATGTCCTCCTCTTCATTATCTTCTCTCATGGCTCCAATAGGCTCCGTGTTGTCATCATACTTGTGTCGGAGATTGTTTTTGTAGCTTTCGCTGGCCTTCTCGTCATCATTTTTACCCACACTTTTGCGATGAGGTCACTCATCATTGGAATCTTGTGCGTGTTCTTTGGTACAATGATGTATGCCGCTCCTTTATCCGTCATGAAACTGGTGATCCAGACAAAAAGTGTGGAGTTCATGCCTCTTTTCCTTTCCCTTGCTTCTTTCTTCAACGGCCTTTGCTGGACTACCTACGCCCTCATCCATTTTGATCTCTACATCACTATTCCGAACGGGCTTGGAGTGCTATTTGCAATGGCTCAGTTAGTGTTACACATCATGTATTACAAAACAACGAAAGAACAGATGGAAGCGCGGAAGAGGAAGACCGAGACAGCTCTCTCAGAAGTGGTCGTGTTCGGAGATAATAACAACAAGGTTGGCAAGGGGTCACAGAACGGCGTGGCCACTCACCCATAG

> HcSWEET4b

ATGGTTAATACCGAAACGATTAGGACGGTTGTTGGCATTATAGGGAATGTGATATCATTGGGGTTGTTCCTGTCACCAGCGCCAACATTTGTTGGAATAATCAAGAAGAAAGATGTGGAGCAATTCTCACCAGTTCCATATCTTGCCACCCTCCTCAACTGCATGTTGTGGGTTCTGTATGGGCTCCCAATTGTGCACCCTGACAGCACTCTGGTCATAACCATCAATGGTGCAGGGGTTGTGATTGAGCTCATCTACATTGCAATCTTTGTCATCTTTAGTGATGGCAAGAAGAGGCTCAATGTGTTCCTCATCTTTATTGGTGAGGTCATCTTCACCTTCACCTTCGGCGTGCTTGTCATCGAGCTCCTCCACACCACCACTCGCCGATCAACCCTTGTCGGAATTCTCTGCGTCATCTTCTGCATTATGATGTATGTCGCGCCATTGTCCGTCATGAGAATGGTTATAAAGACCAAGAGTGTAGAATACATGCCTCTCTTCATCTCTGTTGCATCCTTCTGCAACGGAGCTTGTTGGACTGTCTATTCCCTCCTCAAATTTGACCTTAACATTCTTATCCCCAACGGTATCGGCCTAGTGTTCTCGGTAGTTCAGCTCATACTGTATGCTGTCTTCTACAAATCAACGCAACGATTACTAGAGGCGAGGAAGAAGGCGGAAGTTGGCATGACCGGTATGGGACAAGCTGACAAACTCAGCGATGTAGTCTAG

> HcSWEET4c

ATGGTTAATACCGAAACCATTAGGACGATTGTTGGCGTTGTAGGGAACGTGATATCATTTGGGTTGTTCTTGTCACCAGTGCCAACCTTTGTTGGAATAGTCAAGAGGAAAGATGTGGAGCAATTCTCACCAGTTCCATACCTTGCCACCCTCCTCAACTGCATGCTGTGGGTTCTGTATGGTCTCCCAATTGTGCACCCTGACAGCACTCTGGTCTTAACCATCAATGGAGCAGGGGTTGTGATTGAGCTCATCTACATTGCAGTCTTTATCACCTTTTGTGATGGCAAGAAGAGATTCAATGTGATCCTCATCTTTCTTGGTGAGATCATCTTCACCGTCACCTTCGGCGTCCTTGTCATCGAGCTCCTCCACACCACCACTCGCCGGTCCACCGTCGTCGGAATCCTCTGCGTCATCTTCTGCATTATGATGTACGTCGCGCCACTGTCGGTCATGAGAATGGTTATAAAGACCAAGAGTGTAGAATACATGCCTCTCTTCCTCTCTGTTGCATCCTTCTGTAATGGAACTTGTTGGACTGTCTATTCTCTCCTCAAATTTGACCTCAACATTCTTATCCCCAATGGTATCGGTCTACTGTTCTCAGTAGTTCAGCTCATACTGTATGCTGTCTTCTACAAATCAACGCAACAAATACTAGAGGCGAGGAAGAAGGCAGAAGTTGGCATGACCGGTATGGGACAAGCTGACAAAATCAGCAATGCAGTCTAG

> HcSWEET5

ATGGTTTCGGCAGACACTATCCGAACTTGTGTTGGAATTCTAGGAAATGTGATAGCTCTGGTCCTATTTTTGTCACCAGTGCCAACTTTTTACAGGATTTGGAAGAAAGGTTCAGTTGAGCAATTCTCAGCAGTGCCATACCTGGCAACTCTGCTCAACTGCATGCTTTGGGTGGTGTATGGCCTCCCTTTGGTTCACCCACACAGCACTTTGGTCTTAACCATCAATGGCTCTGGGTTGGTTATTGAGCTCTCCTATGTCCTCCTCTTCATTATCTACTCTCATGGCTCCAATAGGCTCCGCGTTGTCGCCATACTTGTGTCGGAGATTATTTTTGTAGCACTCAGTGGCCTTCTTGTCATAATTTTTACCCACACTATCGCGATGAGGTCATTGATCATTGGAATCTTGTGTGTGTTCTTCGGTACCATGATGTATGCCGCTCCCTTATCCGTCATGAAACTGGTGATCCAGACGAAAAGTGTGGAGTTCATGCCTCTTTTCCTTTCACTCGCTTCTTTCTTCAACGGCCTTTGCTGGACTACCTACGCCCTCATCCGTTTCGATCTCTACATTACTATTCCGAACGGGCTTGGAGTGCTGTTTGCAATGGCTCAGCTAGTGTTACACATCATTTACTATAAAACAACCAAAGAACAGATGGAAGCGCGGAAGAGGAAGGCCGAGACAGGTCTCTCAGAAGTGGTCGTGTTTGGAGATAATAACAATAAGGTCGGCAAGGGGTCACAGAATGGCGTCCCCTAG

> HcSWEET6

ATGGTTTCTGCAGATACTATCCGCACTGTTGTTGGGATCCTAGGAAATGTCATATCACTCATCTTGTTTCTATCTCCTGCGCCAACATTTATTAGGATATGGAAGAAGGGGTCAGTGGAGCAGTTCTCACCAGTCCCATACCTCGCAACTCTCCTTAACTGCTTGCTTTGGGTGGTCTATGGCCTCCCTTTAGTTCACCCGCATAGCACTCTTGTTTTAACTATTAATGGTTCCGGGGTGGCTATAGAGCTCTTCTACGTTCTCCTCTTCATTATCTACTCCCAGGGCTCCAAAAGACTCCGAGTGCTAATCATTTTGGTATCTGAAATTGCTTCCGTGATGCTTGTTGGCGTTCTAGTTATCGTTTTCGCCCACACACTGGCAATGAGGTCATTGATCATCGGAATCTTGTGTGTGTTCTTCGGGACAATGATGTATGCTGCTCCATTGTCAGTCATGAAATTGGTTATCCAAACCAAAAGCGTGGAGTTCATGCCTCTCTTCCTTTCTCTTGCTTCTTTCTTCAATGGTCTTTCTTGGACTGCTTATGCTCTCATCCGCTTCGATCTCTTTGTTACTATTCCGAATGCGCTTGGAGTTATATTTGCAGTGGCACAGCTGGTGTTACATATTATGTACTACAAATCAACCAAAGAGCAGATGGAAGCACGAAAGAGGAAGGCCGAAACCGGACTTTCTGAAGTAGTTGTTCATGCAGACAGTAACACAAAGGTCAACGATGCACCGAATTAA

> HcSWEET11

ATGGCAGGCTTATCGTTCGACCACCCATGGGTCTTAATCTTTGGACTTCTTGGCAACATACTCTCAGGCATGGTCTACCTCTCCCCAGTGCCAACGTTTCGTCGGATTTGTAGGAAGAAATCGACAGAAGGGTTCCAGTCATTCCCTTACTGTGTGGCATTGTTCAGTGCCATGCAACTGATCTACTATGCAATTATCAAGACAAACACTTACCTACTCATCACCATTAACACTGCTGGGTGCGTCATTGAGTCTGCCTACATCATTATATATCTGATCTATGCACCCAAGAAGGCTAGGATTAACACAGTGAAAACAATGTTACTGCTGAATGTGGGGTTATTCTCTGCAATCGTTCTCATCAGTTTCTTGTGCTTCAAGGGCGCTGACCGAGTTAAAGTTGTAGGTTGGATCAATATGACCTTCTCAGTCAGTGTCTTCGCAGCTCCTTTGAGCATTATTAGGCTCGTTATTCGCACTAAGAGTGTGGAATTCATGCCCTTCTTCCTATCGTTCTTCCTCACCATGAGTGCCATCGCGTGGTTCTTCTACGGCCTTCTCACCCGAGACATATATGTCGCATTTCCAAACATACTGGGGTTCACCTTCGGATGCATTCAGATGATTCTTTACATTGTGTACAAGGATGCCAAAAAAGGGAAAGAGGACATTGAAGGTAAGCTACCTGAAGAAGTACTTGATGATACTGCAAAGCCGAGCCCGATGGGAGTGGAAGTTAGTAAGGCTGAAACTGCAGAGGATAATACAAGCAGGGTGGATAACGCAAGCCAAGTGTGA

> HcSWEET13a

ATGGCAGGACTTTCATTAGACCACCCTTGGGCTTTTGCCTTTGGTCTCCTAGGCAACTTGATCTCATTCATGGTGTTACTTGCTCCAATTGCTATGTTATGGATCTACTATGCATTCGTCAAGACAAATGAATACCTTCTCATCACCATCAACACATTTGGATGCTTCATTGAGACTCTATACATCGTCATGTATCTCACATATGCTCCTAGAAAAGCTAAGGTCAACACAGCAAAAATTTTCTTGCTTTTGAATGTAGGACTTTTCTCCTTGATAGTTCTTGCCACTATTTTCCTCTCAAAGGGTGCCAACCGCCAAAAGCTTCTCGGGTGGATCTGTGTGGGCTTTGCAGTCAGCGTATTTGCTGCACCTTTGAGCATAATTAAGCAAGTCATAAAAACTAAGAGTGTGGAGTACATGCCATTTTCTCTATCCTTCTTCCTCACATTGAGTGCAGTTGTGTGGTTCGCCTATGGTCTACTTATAAAGGACATCTATGTCGCGATACCAAATATATTGGGATTCGTCTTTGGAATAATCCAAATGGTGTTGTACATAATCTACAATGATGTCAAGGTACTAAAAGAGGAGCTCAAGATAGGTGAGTCAACTGAAGGAAAGATGGGGGAAATTACTGTGGAAGTTGAAATAGAAAAGGGGGACACTACTGTCAAGGGAGGGAAAAAGATGTCAATGGAGAAGGCAAAGATGGGTTTAAAAGATGGGGTTGAGATGTGTCAAGTTTGA

> HcSWEET13b

ATGGCAGGATTATCCTTAGACCACCCTTGGGCTTTCGCCTTCGGCATTCTAGGCAACTTGATCTCATTCATGGTGTGCCTTGCCCCAATACCAACATTCTACCGGATATACAAGAGTAAATCAACTCAAGGGTTCCAATCAGTGCCATATGTGGTTGCACTGTTTAGCGCAATGCTATGGATCTACTATGCGTTAATCAAAACCAATGAATACCTTCTCATCACCATCAACTCAGTTGGATGTGTCATTGAGACTATATACATCATCATATTTATCACCTACGCCTCGAAGAAAATTAAGATCCACACTGTAAAACTGATGTTGCTCCTGAATGTGGGACTATTTGCTTCGATTGTTCTATCGACGCTCCTACTGACAAAGGGTCCCACGCGTGTAACAGTTGTCGGGTGGATGTGTGTAGGCTTCGCAGTCAGCGTTTTCGCTGCCCCTTTGAGCATAATTAGGCAAGTTATGAGGACAAGGAGTGTGGAATTCATGCCCTTCTCCCTTTCCTTCTCCCTCACACTAAGTGCAGTTGCTTGGTTCTCCTATGGTTTATTAACAAAGGACATTTACGTCGCGATTCCCAACATATTAGGATTCAGCTTTGGAGTAGTCCAAATGGTTCTCTACATAATCTACAAGGATGCAAAGGAGTTAGTGAAAGATGACAAGGTGGATGAGACAGAACATGTTGTAATATCCATTGCAAAGCTTAATGAGAGCAGAGTTGCACCCCAAGTTAGCGCGAGCGAACAAGAATTAGTGCAAGCAGCAGAGGAGAATAACAAAGAGGAGGAGGAGGAGGGGAAAGAAATGTTAGAAGTAGAAGGCCAAAATGCAATTGAAATGAGTAGTCCAGTTTAG

> HcSWEET13c

ATGGCAGGATTATCTTTAGACCACCCTTGGGCTTTCGCCTTCGGCATTCTAGGCAACTTGATCTCATTCTTGGTGTACCTTGCCCCAATACCGACATTCTACCGTATATACAAGAGTAAATCAACTCAAGGGTTCCAATCAGTGCCATACGTTGTTGCTTTGTTCAGTGCAATGCTATGGATCTACTATGCATTCATCAAAACCAACGAATACCTTCTCATCACCATCAACTCAGTTGGATGTGTCATTGAGACAGTATATATCATCATGTTTATCACATACGCCTCAAAGAAAATTAAGATCCACACTGTAAAATTGATGTTGCTTCTGAACGTGGGACTATTTTCTTCGATCGTTCTATCAACGCTCCTACTGGCCAAGGGTCCCAAGCGTGTAACAGTTCTCGGGTGGATGTGTGTAGGCTTCGCAGTCAGCGTTTTCGCTGCCCCTTTGAGCATAATTAGGCAAGTTATTAGGACAAGGAGTGTGGAATTCATGCCCTTCTCCCTCTCCTTCTCCCTTACATTGAGTGCAGTTGTTTGGTTCTCCTATGGTCTATTAACAAAGGACATTTACGTTGCGATTCCCAACATATTAGGATTCAGCTTTGGAGTAGTCCAAATGATTCTGTATATAATCTACAAGGATGCAAAGGAACTAGTGAAAGATAACAAGTTGGATGAGAAAGAACAGGTTGTAATATCCATTCCACGGCTTAATGAGAGCAGAGTTGCACCCCAAGGCGATCAAGAATTAGTGAAAGCAGGGAAGGAGAACAACAAAGAAGAGGAGAAGGAGATGGTAGAAGTAGAAGGCCAAAATGGAATTGAAATGAGCCCAGTTTAG

> HcSWEET13d

ATGGTGTATCTGGCCCCCATACCAACCTTCTACCGGATTTACCGAAAGAAATCAACTGAAGGATTTCAATCGTTGCCCTACGTGGTTGCACTGTTCAGTGCGATGCTATGGATCTACTATGCATTCATCAAGACCGATGAGTACCTTCTCATCACCATTAACACTGTTGGATGTGTCATTGAGACTATATACATCATCATGTTTCTTGCCTACGCTCCGAAGAAGGTCAAGATCCATACCGTGAAAATGATCTTGCTCCTGAATGTGGGACTGTTTTCTTTGATCGTGCTCTCAACTCTCCTACTCTCAAAAGGTGCCGACCGTGTAAAAGTTCTCGGGTGGATGTGTGTAGGCTTTGCAGTCGGTGTTTTCGCAGCCCCTTTGAGCATAATTAGGCAAGTTATCAGAACAAAGAGTGTGGAGTTTATGCCCTTCTCGCTGTCCTTCTCCCTTACACTGAGTGCAATTGTTTGGTTCTCCTATGGTCTACTGACAAAGGACATTTATGTCGCGATACCCAATATACTTGGATTTAGCTTTGGAACAGTCCAAATGGTTCTATACCTAATCTACAAGGATGCAAAGGATTTGATGATAAACGAGGACAAGTTGCCCAACACCAAAGTGATCTCACAGAACAACGTTAATACCGAAGATACTAAGCTCGATGAGGAGAAGAAAGGCTATCATGAGGACAAGAAGGACATGGTAGAAGGGAAGGATGAAATTGAATTGAGCCCAGTGTAG

> HcSWEET14a

ATGGCAGGGCTTTCACTTGATCATCCTTGGGCTTTTACATTTGGCCTACTAGGCAACTTGATCTCATTCATGGTTTACCTTGCTCCAATACCGACATTCCACCGCATATACAAGAAGAAATCGACTGAAGGATTCCAATCAGTTCCCTACGTGGTTGCGCTGTTCAGCGCCATGCTGTGGATCTACTACGCATTCATCAAATCAAACGAATACCTTCTCATTACCATCAACTCGCTTGGCATAGTGATTGAGACCGTCTACATCATCATGTTTATCGCCTATGCCCCAAAGAAGGCCAAGGTCTACACAGCAAAAATAATTATGGGTTTGAATGTGGGCCTCTTCTCCTTGATTGTTCTTTCCACTCTTTTACTCACAAAAGGAGCCCACCGTCAGAAACTTCTTGGATGGATGTGTGTGGGATTTGCGGTCAGTGTCTTTGCGGCTCCTTTGAGCATAATTAGGCAAGTCATCAGAACCAAGAGCGTCGAGTTCATGCCATTCTCACTATCCTTCTTCCTCACCTTGAGCGCAATCGTCTGGTTCTCCTACGGTCTACTTATAAAGGACATCTATGTCGCGATTCCAAATATATTGGGGTTCAGCTTTGGAGTAATCCAAATGATATTGTACATAATCTACAAGGATGTCAAGGTGCTTAAAGAAGAGCTCAAGCTACCAGAGGACCTTGCAACCATGGCAAAGCTAGGCGAGATCATGATCGAAGCACAGGGGATCGCCGTGGAAATTGAAGTGAAGGAGGAGAAGATGAAGAGGGTGAAGGAAGAAGAGAAGAAAATTACTGGAATCGAGCAGGCTATTAAAGGGGAGAATTAA

> HcSWEET14b

ATGGCAGGGCTTTCACTTGATCACCCTTGGGCTTTTACTTTTGGCCTCCTAGGCAACTTGATCTCATTCATGGTTTACCTTGCTCCAATACCGACATTCCACCGCATATACAAGAAGAAATCAACAGAAGGATTCCAATCAGTTCCCTACGTGGTCGCGCTCTTCAGTGCCATGCTGTGGATCTACTACGCATTCATCAAATCAAACGAATACCTTCTCATTACCATCAACTCGCTTGGCATAGTGATTGAGACTGTCTACATCATCATGTTTATCGCCTATGCCCCAAAGAAGGCCAAGGTCTACACAGCAAAAATAATCATGGGTTTGAATGTGGGCCTCTTCTCCTTGATTGTTCTATCCACTCTTTTACTCACAAGAGGAGCCAACCGTCAGAAACTTCTTGGATGGATGTGTGTGGGATTTGCGGTCAGTGTCTTTGCTGCTCCTTTGAGCATAATTAGGCAAGTCATCAGAACCAAGAGCGTCGAGTTCATGCCATTCTCACTATCCTTCTTCCTCACCTTGAGCGCAATCGTCTGGTTCTCCTACGGTCTACTTATAAAGGACATCTATGTCGCGATTCCAAATATATTGGGGTTCAGCTTTGGAGTAATCCAAATGATATTGTACGTAATCTTCAAGGATGTCAAGGTGCTTAAAGAAGAGCTCAAGCTACCAGAGGACCTTGCAACCATGGCAAAGCTAGGCGAGATCATGATCGAAGCAGAGGGGATCGCCGTCGAAATTGAAGTGAAGGAGGAGAAGATGAAGAAGGTGAAGGAAGAGAAGAAAATTACTGGAATCGAGCAGGCTATTAAAGGGGAGAAGGATGAGGTTGAGATGTGCCAAGTGTAG

> HcSWEET14c

ATGGCAGGGCTTTCACTTCATCACCCTTGGGCTTTTACTTTTGGCCTCCTAGGCAACGTGATCTCATTCATGGTTTACCTTGCTCCAATACCGACATTCTACCGCATATACAAAAAGAAATCGACTGAAGGATTCCAATCAGTTCCCTACGTGGTTGCACTGTTCAGTGCCATGCTGTGGATCTACTACGCATTTATCAAAACAAACGAATACCTGCTCATTACCATCAACTCTTTTGGCATCGTGATTGAGACCGTCTACACCGTCATGTTTCTCGCCTATGCCCCAAAGAAGGCCAAGGTCTACACAACAAAACTTATCATGGGTTTGAATGTGGGCCTCTTCTCCTTGATTGTTCTGTCCACTCTTTTCCTCACAAAAGGAGCCAAACGTCAGAAACTTCTTGGATGGATTTGTGTGGGATTTGCGGCCAGTGTCTTTGCTGCTCCTTTGAGCATAATTAGGCAAGTCATCAGAACCAAGAGTGTCGAGTTCATGCCATTCTGGCTATCCTTCTTCCTCGCCTTGAGCGCAATGGTCTGGTTCTCCTATGGTCTACTTATAAAAGACATCTATGTCGCGATACCAAATATATTGGGGTTCAGCTTTGGAGTAGTCCAAATGGTGTTGTACATAATCTACAAGGATGTGAAGGTGCTTAAAGAAGAGCTCAAGCTACCAGAGGACCTTACAACCATGGCAAGGCTAGGCAAGAATGTGATCGAAGCACAGGAGATCACCGCAGAAATTGAAGTGAAGGGGGACAAGAAGAAGGTGAAGGAAGAGGAGAAGAAGATTATTGGAATGGAGAAAGCTATTAAAGGGGAGAAGGATGATGTTGAGATGTGCCAAGTGTAG

> HcSWEET15

ATGTGTAGCATGGCGGAGGAACACCACTGGGCCTTTGCATTCGGCATCCTAGGTAATATCATCTCCTTCATGGTGTACCTAGCTCCACTGCCTACATTTCTCCGAATATACAAAAAGAAATCAACGCAAGGGTTCCAATCCATCCCTTACGTCGTTGCACTCTTCAGCGCGTCTTTGTGGATATACTATGCCATCCTCAAGAGCACCAACACATTTCTTCTCATCACCATTAACGCCGCTGGTTGTGTAATAGAGGCTGGCTACATCATCTTCTACCTCATTTACGCACCAAGGAATGCTCGGATATACACAGCGAAACTACTCCTGCTCTTGAATGTAGGCCTTTTCGGTTTGATTATTCTCCTCACTCTCCTACTATCAACTGGGTCAGGTCGGGTAGTAACCCTTGGTTGGATCTGTGTGGGCTTCTCAACCTCTGTATTTGTCGCACCATTAAGCATTATCAGACTTGTCATCCAAACAAAGAGCGTCGAGTTCATGCCCTTCTCCTTATCGTTTTCTCTCACCCTCAGCGCTGTCGTTTGGTTCTTCTACGGATTTCTCTCCCATGATATATACATTGCGCTCCCCAATGTACTAGGATTCATCTTTGGTGTGATCCAAATGCTGCTATATGCAATGTACATGAGCGAAAACAAACCAAAGATTGTTGAACAACATCATGCAATGCACGAACAAATCATAACCATTGCGGAGCTAGGGGTAGTGCTCGGGTTGGAGGTTGACCTGGTTGACACAGCGACGGACGCAAGCAAGGAGAATATTGGGGGACAAGGGAATGAGTGTGAGTGTGAGTGTGAGCTAACCAACATTCAAGGGCAAGAGGGGATTAATGAGATGATCCTTTGA

> HcSWEET16

ATGGCTGCTCCAAGCTTGATAGTTGGAATTGTGGGGAATGTGATCTCCATACTAGTATTTGCCTCTCCCATAACGACGTTCTGGAGAATAGTGAAGAAGAAGTCGACGATGAACTATGAAGGGTTACCATATGTGACGACTCTGTTGTGCACATCTATGTGGACATATTATGGGCTACACAAGCCCGGGGGTCTTCTGATTGTGACTGTAAATGGTGCTGGGAGTGTGATGCAGTCGGTTTATGTTGTGCTGTTCCTCATCTACGCTCAAACAAGCACACGAATTAGGGTTGGCAGATTGGTGGGTATATTGAATGTAGGGGTGTTTGGGATGGTGATATTGGTGACGAGTTTAGCGCTTCACGGGAATTTGAGGCTGCTGGTTGTTGGGTGCATGTGTGCTGGCCTCACCGTCGGCATGTATGCTGCACCTATGGCTGTTATGAGACTAGTTGTGCAGACTAGGAGTGTTGAATACATGCCATTCTCACTCTCCTTCTTCCTTTTTCTCAATGGTGGTGTTTGGGGTGCCTATGCCTTTTTGGTGAAAGACTTCTTTATTGGGATTCCAAATGTCATAGGATTTGTATTGGGCACGGCGCAATTGATCCTTTATGCAACTTACAGAAAGAAGTCACCAGTTGCAAAGGAAGTGGACTTGGAAATGGGGAGAATGGACCAAATTGCAGAGCAGAAGCTGAAAATGGCGTCTAACGCACAGGATAAAAATCATTTGCACAGAGGAGCTAGCTTGCCAATGAAGAGATCAGTTTCTCGCCAGCGAAGCTTAACCAAGATTGTCAAGTCATTGTCATTACCTCCTTATGAGAATCCTGACTGGAGCCTTGATGATCTTGATAACCATTCTGAGGTCGAACATCCTAAGCAAATATTAGGATAA
