# Supplementary material for: Genome-wide analysis of the SWEET gene family in Hemerocallis citrina and functional characterization of HcSWEET4a in response to salt stress
Source: BMC Plant Biol. 2024 Jul 11;24:661. doi: 10.1186/s12870-024-05376-y (PMC11238388; doi:10.1186/s12870-024-05376-y)
Supplement: Supplementary file 3 — Supplementary Material 3 [file 12870_2024_5376_MOESM3_ESM.docx]

**Supplementary Figures and Tables:**

**Supplementary Figure S1** Tertiary structure predictions of HcSWEET proteins

**Supplementary Table S1** Secondary structure and subcellular localization predictions of HcSWEETs

**Supplementary Table S2** Primers for PCR used in this paper

**Supplementary Table S3** FPKM average value of three biological replicates from night lily tissue-specific RNA-seq data

**Supplementary Table S4** FPKM average value of three biological replicates from night lily RNA-seq data under drought stress

**Supplementary Table S5** FPKM average value of three biological replicates from night lily RNA-seq data under salt stress


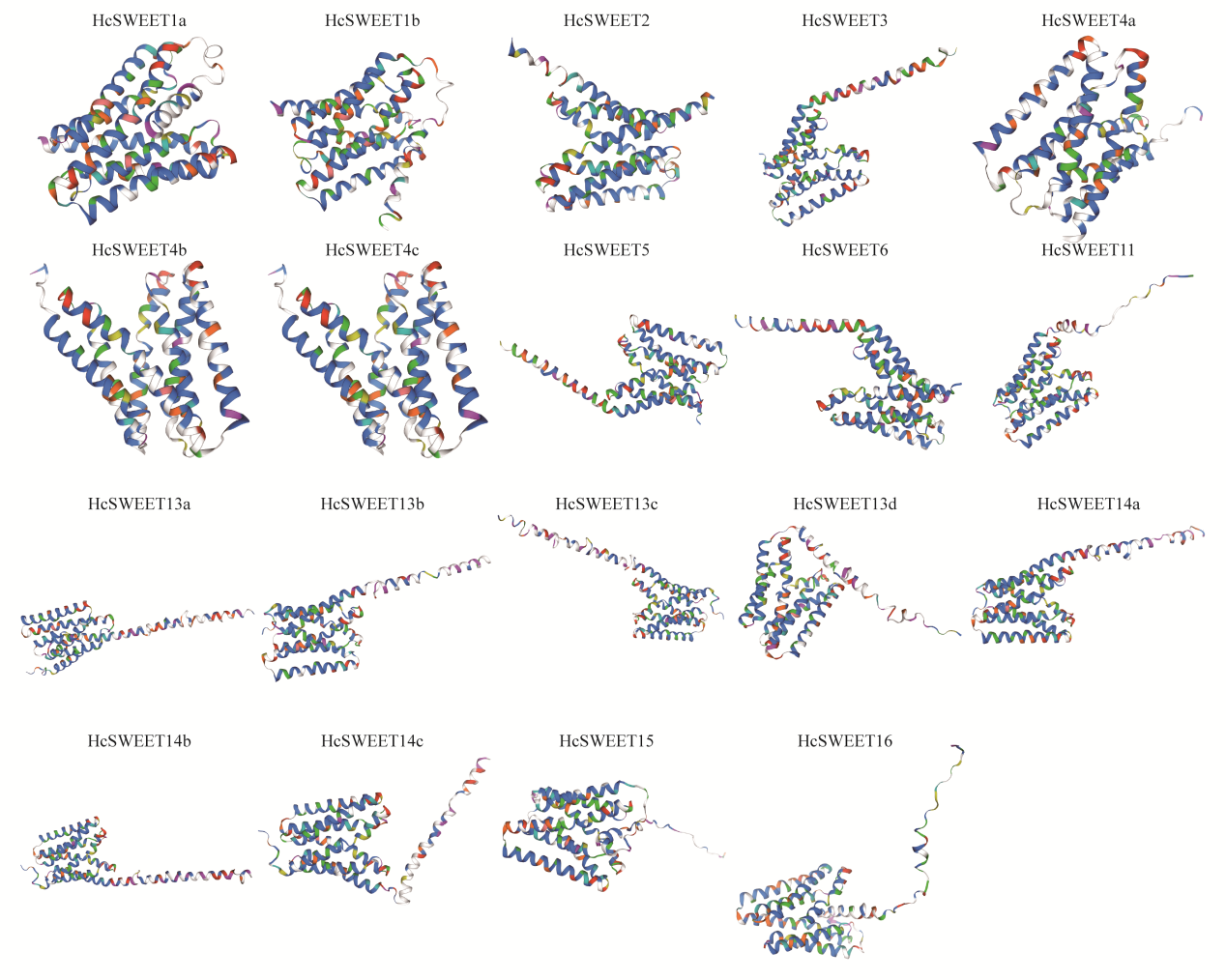


**Supplementary Figure S1** Tertiary structure predictions of HcSWEET proteins. Protein IDs are listed at the edge. Different colors indicate various helical and folding regions based on seven transmembrane helices (TMs).

**Supplementary Table S1** Secondary structure and subcellular localization predictions of HcSWEETs

| Protein  symbol | α-helix  (%) | Extended strand  (%) | β-turn  (%) | Random coil  (%) | Subcellular localization |
| --- | --- | --- | --- | --- | --- |
| HcSWEET1a | 44.67 | 17.62 | 4.51 | 33.20 | Chloroplast |
| HcSWEET1b | 37.01 | 18.90 | 1.97 | 42.13 | Chloroplast |
| HcSWEET2 | 47.60 | 20.09 | 2.62 | 29.69 | Plasma membrane |
| HcSWEET3 | 44.54 | 21.43 | 2.94 | 31.09 | Plasma membrane |
| HcSWEET4a | 39.30 | 20.62 | 3.11 | 36.96 | Plasma membrane |
| HcSWEET4b | 43.85 | 22.54 | 3.69 | 29.92 | Plasma membrane |
| HcSWEET4c | 38.52 | 24.59 | 4.51 | 32.38 | Plasma membrane |
| HcSWEET5 | 36.22 | 23.62 | 2.76 | 37.40 | Plasma membrane |
| HcSWEET6 | 40.00 | 21.20 | 3.60 | 35.20 | Chloroplast |
| HcSWEET11 | 37.36 | 20.00 | 3.77 | 38.87 | Chloroplast |
| HcSWEET13a | 49.60 | 16.13 | 0.81 | 33.47 | Vacuole |
| HcSWEET13b | 48.96 | 17.01 | 3.47 | 30.56 | Chloroplast |
| HcSWEET13c | 43.77 | 18.15 | 3.20 | 34.88 | Chloroplast |
| HcSWEET13d | 39.92 | 20.16 | 1.61 | 38.31 | Plasma membrane |
| HcSWEET14a | 56.12 | 16.19 | 3.60 | 24.10 | Chloroplast |
| HcSWEET14b | 54.74 | 18.60 | 3.86 | 22.81 | Chloroplast |
| HcSWEET14c | 49.47 | 19.30 | 3.16 | 28.07 | Chloroplast |
| HcSWEET15 | 44.98 | 16.26 | 3.11 | 35.64 | Cytoplasm |
| HcSWEET16 | 48.16% | 16.39% | 2.01% | 33.44% | Chloroplast |

**Supplementary Table S2** Primers for PCR used in this paper

| Primer Name | Sequences（5' to 3'） | Usage |
| --- | --- | --- |
| qHcSWEET1a-F | CTGTGCGGGACCTCTTGG | RT-qPCR |
| qHcSWEET1a-R | GCACTAATCCAAGCGCGC |  |
| qHcSWEET2-F | ATTCAGTTGGTGCCGCGT |  |
| qHcSWEET2-R | TGAGGGCAAAAGCACCGA |  |
| qHcSWEET4a-F | GAACGGGCTTGGAGTGCT |  |
| qHcSWEET4a-R | CCTCTTCCGCGCTTCCAT |  |
| qHcSWEET4b-F | ACCGACATTCCACCGCAT |  |
| qHcSWEET4b-R | CACTGAAGAGCGCGACCA |  |
| qHcSWEET5-F | GAACGGGCTTGGAGTGCT |  |
| qHcSWEET5-R | ACCTGTCTCGGCCTTCCT |  |
| qHcSWEET13c-F | CTGGCCAAGGGTCCCAAG |  |
| qHcSWEET13c-R | GCTCAAAGGGGCAGCGAA |  |
| qHcSWEET14a-F | AGGAGCCCACCGTCAGAA |  |
| qHcSWEET14a-R | TGGCATGAACTCGACGCT |  |
| qHcSWEET14b-F | ACCGACATTCCACCGCAT |  |
| qHcSWEET14b-R | CACTGAAGAGCGCGACCA |  |
| qHcSWEET14c-F | GGCCAGTGTCTTTGCTGC |  |
| qHcSWEET14c-R | GACCATTGCGCTCAAGGC |  |
| qHcACTIN-F | AGCCCCTTGTTTGCGACA |  |
| qHcACTIN-R | CACCAGTGTGGCGAGGTC |  |
| qClACTIN-F | CCATGTATGTTGCCATCCAG |  |
| qClACTIN-F | GGATAGCATGGGGTAGAGCA |  |
| HcSWEET4a-sub-F | GCTCTAGAATGGTTTCAGCCGACACCAT | Subcellular localization |
| HcSWEET4a-sub-R | GGGGTACCTGGGTGAGTGGCCACGCCGT |  |
| HcSWEET5-sub-F | GCTCTAGAATGGTTTCGGCAGACACTAT |  |
| HcSWEET5-sub-R | GGGGTACCGGGGACGCCATTCTGTGACC |  |
| p1300-seq-F | GCCTTTTCAGAAATGGATAAATA |  |
| p1300-seq-R | CGCCGTAGGTCAGGGTGGTC |  |
| HcSWEET4a-xg-F | CGCGGATCCATGGTTTCAGCCGACACCAT | Genetic transformation |
| HcSWEET4a-xg-R | GGGGTACCTGGGTGAGTGGCCACGCCGT |  |
| P35S-F | GACGCACAATCCCACTATCC |  |
| 1305.4-check-R | TTAGGTTTACCCGCCAAT |  |

**Supplementary Table S3** FPKM average value of three biological replicates from night lily tissue-specific RNA-seq data

| Gene ID | Tender Root | Mature Root | Bud | Tender Leaf | Mature Leaf | Tender Scape | Mature Scape |
| --- | --- | --- | --- | --- | --- | --- | --- |
| *HcSWEET1a* | 1.8670673 | 90.2226217 | 42.14409 | 30.7103543 | 2.278777 | 59.8234353 | 3.57961033 |
| *HcSWEET1b* | 0.143905 | 1.04156433 | 27.56680 | 33.5987337 | 26.913148 | 5.882552 | 2.34654567 |
| *HcSWEET2* | 11.091891 | 48.689418 | 4.552146 | 6.424882 | 5.5893487 | 8.73187033 | 3.348905 |
| *HcSWEET3* | 0 | 0 | 0 | 1.36900333 | 0.6035683 | 5.412528 | 13.0360647 |
| *HcSWEET4a* | 139.71979 | 47.917094 | 2.513460 | 0.561038 | 0.414709 | 15.49343 | 145.442460 |
| *HcSWEET4b* | 32.477595 | 5.01271033 | 0 | 15.0007847 | 0.052466 | 1.58415233 | 0 |
| *HcSWEET4c* | 0 | 0 | 12.60189 | 46.7420277 | 0.281723 | 9.40262433 | 0.60185367 |
| *HcSWEET5* | 13.087190 | 12.0885027 | 27.22815 | 1.33623 | 0 | 0.70694433 | 0.39984533 |
| *HcSWEET6* | 0.045479 | 0.055963 | 26.79041 | 1.52556133 | 0 | 8.28856067 | 4.838966 |
| *HcSWEET11* | 0.0459973 | 2.69694967 | 39.18573 | 0 | 0 | 0.174989 | 0.05360867 |
| *HcSWEET13a* | 0 | 0.03303067 | 47.16362 | 0 | 0.5416673 | 0.37203667 | 0.34542567 |
| *HcSWEET13b* | 1.097377 | 1.65303633 | 0.851293 | 0 | 0.206285 | 6.178426 | 0.49330867 |
| *HcSWEET13c* | 141.46490 | 8.00015633 | 0.096034 | 0 | 0 | 3.46856433 | 91.4634627 |
| *HcSWEET13d* | 63.689034 | 2.748971 | 0.439209 | 0.06938267 | 0.2815543 | 0.073024 | 0.04237267 |
| *HcSWEET14a* | 36.893785 | 12.1907663 | 0.069649 | 0.02375867 | 0 | 0 | 2.80844433 |
| *HcSWEET14b* | 52.782615 | 2.53221067 | 0.148876 | 0 | 0 | 0.08431133 | 0.206139 |
| *HcSWEET14c* | 318.81442 | 91.912263 | 56.31336 | 0 | 0 | 0.22219967 | 1.74402233 |
| *HcSWEET15* | 0.78124 | 6.41073167 | 0 | 0 | 0 | 0.821561 | 0 |
| *HcSWEET16* | 0.458055 | 1.196481 | 2.8379073 | 27.11636067 | 4.6009147 | 35.2429033 | 11.7334773 |

**Supplementary Table S4** FPKM average value of three biological replicates from night lily RNA-seq data under drought stress

| Gene ID | Drought-0 h | Drought-24 h | Drought-48 h | Drought-72 h | Drought-108 h |
| --- | --- | --- | --- | --- | --- |
| *HcSWEET1a* | 105.889465 | 171.96994 | 86.770912 | 286.738342 | 223.019058 |
| *HcSWEET1b* | 0.290454 | 0.670191 | 1.035484 | 0.511883 | 0.153097 |
| *HcSWEET2* | 41.165115 | 28.614729 | 33.624657 | 26.218655 | 27.2794 |
| *HcSWEET3* | 0 | 0.098322 | 0.434135 | 0 | 2.314281 |
| *HcSWEET4a* | 24.554419 | 300.483101 | 249.879201 | 255.028982 | 274.953938 |
| *HcSWEET4b* | 31.673723 | 43.692382 | 12.766745 | 42.741901 | 34.616063 |
| *HcSWEET4c* | 4.146312 | 0.162755 | 0.074613 | 0.253824 | 4.408318 |
| *HcSWEET5* | 1.647796 | 33.408318 | 40.397228 | 30.848701 | 9.210407 |
| *HcSWEET6* | 0.86341 | 1.46254 | 17.748344 | 2.556003 | 0.371717 |
| *HcSWEET11* | 0.089786 | 0.048643 | 17.220934 | 0.505033 | 0.297491 |
| *HcSWEET13a* | 0.04536 | 0 | 0 | 0 | 0.050457 |
| *HcSWEET13b* | 2.622307 | 1.255225 | 8.176724 | 1.753936 | 0.654212 |
| *HcSWEET13c* | 2.730818 | 99.5541 | 63.219551 | 32.453941 | 6.730563 |
| *HcSWEET13d* | 1.395298 | 4.265241 | 24.934669 | 22.107519 | 14.004094 |
| *HcSWEET14a* | 7.253256 | 9.226583 | 0.728151 | 1.137434 | 1.395595 |
| *HcSWEET14b* | 7.397691 | 3.666586 | 4.855793 | 4.396758 | 0.978926 |
| *HcSWEET14c* | 3.948853 | 25.830627 | 26.402531 | 11.494273 | 4.598904 |
| *HcSWEET15* | 1.260294 | 0.109489 | 0.269376 | 2.690298 | 0.63916 |
| *HcSWEET16* | 0.592739 | 27.371788 | 3.412104 | 4.951363 | 0.775553 |

**Supplementary Table S5** FPKM average value of three biological replicates from night lily RNA-seq data under salt stress

| Gene ID | Salt-0 h | Salt-24 h | Salt-48 h | Salt-72 h | Salt-96 h |
| --- | --- | --- | --- | --- | --- |
| *HcSWEET1a* | 90.080833 | 277.382294 | 118.739471 | 55.356106 | 52.502422 |
| *HcSWEET1b* | 0.298968 | 0.735571 | 1.009664 | 0.409459 | 1.012159 |
| *HcSWEET2* | 35.005714 | 12.70079 | 21.987982 | 15.859769 | 16.078392 |
| *HcSWEET3* | 0 | 0 | 0 | 0 | 0 |
| *HcSWEET4a* | 40.345385 | 90.815624 | 73.231487 | 104.598907 | 50.011986 |
| *HcSWEET4b* | 11.62929 | 140.303718 | 53.219722 | 50.553368 | 22.042568 |
| *HcSWEET4c* | 2.452323 | 1.731412 | 3.408292 | 0.403384 | 1.271804 |
| *HcSWEET5* | 1.276842 | 16.092419 | 18.039076 | 29.525354 | 9.29406 |
| *HcSWEET6* | 0 | 0.092916 | 0.079321 | 0.282038 | 0 |
| *HcSWEET11* | 1.004131 | 0.927296 | 14.744946 | 0.313477 | 17.308889 |
| *HcSWEET13a* | 0.053299 | 0 | 0 | 0 | 0 |
| *HcSWEET13b* | 0.462198 | 0.788481 | 1.162522 | 1.179423 | 2.670183 |
| *HcSWEET13c* | 2.142837 | 2.912288 | 10.203822 | 6.222336 | 6.061832 |
| *HcSWEET13d* | 0.386536 | 0.771638 | 1.487868 | 4.938987 | 3.324219 |
| *HcSWEET14a* | 21.651436 | 5.082366 | 16.648169 | 8.215014 | 13.803571 |
| *HcSWEET14b* | 19.638281 | 22.731327 | 13.556771 | 11.632293 | 8.516203 |
| *HcSWEET14c* | 6.216188 | 2.513687 | 13.453885 | 8.042294 | 12.864455 |
| *HcSWEET15* | 1.153023 | 1.886508 | 3.213803 | 0.346082 | 3.095108 |
| *HcSWEET16* | 1.568933 | 1.231494 | 1.826422 | 1.439667 | 0.624233 |
